# Self-Normalized Cramér-Type Moderate Deviations for the Harris Estimator of a Supercritical Branching Process

**DOI:** 10.3390/e28101145

**Published:** 2026-10-18

**Authors:** Zixuan Jia, Xiequan Fan

**Affiliations:** School of Mathematics and Statistics, Northeastern University at Qinhuangdao, Qinhuangdao 066004, China; 202412492@stu.neuq.edu.cn

**Keywords:** Galton–Watson process, Harris estimator, self-normalized moderate deviations, Berry–Esseen bounds, 60F10, 60J80, 60J10, 62F03

## Abstract

Let ${\left(X_{n}\right)}_{n\geq 0}$ be a supercritical Galton–Watson process with offspring mean *m* > 1, positive variance, and ${E|X_{1}-m|}^{2+\rho }<\infty$ for some 0 < ρ ≤ 1. We establish self-normalized Cramértype moderate deviations for the Studentized Harris estimator. It is shown that there exists a constant *c* > 0 so that the logarithmic relative error in normal approximation is of order *O*(*e*^−*cn*^), uniformly for 0 ≤ *x* ≤ *c*$\sqrt{n}$. The self-normalized Cramér-type moderate deviations hold with condition *p*_0_ = 0, including the Böttcher case *p*_0_ = *p*_1_ = 0. When *p*_0_ > 0, similar results hold conditional on non-extinction or on *X_n_* > 0. Consequently, we obtain moderate deviation principles, Berry–Esseen bounds, and asymptotic confidence intervals. Numerical experiments for bounded and unbounded offspring laws with *p*_0_ = 0, *p*_1_ > 0 examine tail approximation and interval coverage.

## 1. Introduction

Consider a Galton–Watson process ${\left(X_{n}\right)}_{n\geq 0}$ with $X_{0}=1$, defined by

(1) $$X_{n+1}=\sum_{i=1}^{X_{n}}Y_{n+1,i},\;n\geq 0,$$

where ${\left(Y_{n,i}\right)}_{n,i\geq 1}$ are independent and identically distributed with $P\left(Y_{n,i}=r\right)=p_{r}$, r≥0. Let

$$F_{n}=\sigma \left(Y_{k,i}:1\leq k\leq n,\;1\leq i\leq X_{k-1}\right)$$

denote the natural filtration generated by the observed offspring variables. The offspring mean is

(2) $$m=EX_{1}=EY_{1,1}=\sum_{r\geq 1}rp_{r}.$$

Write $\sigma^{2}=\mathrm{Var}\left(X_{1}\right)$ for the variance of $X_{1}$ and $S_{n}=\sum_{k=0}^{n}X_{k}$ for the cumulative population size. Throughout the paper, we assume that m>1 and $0<\sigma^{2}<\infty$.

Large deviations for the Lotka–Nagaev estimator $X_{n+1}/X_{n}$ were studied in [1,2,3,4]; see also [5,6] for multitype processes. Recently, Fan and Shao [7] established self-normalized moderate deviation results for the Lotka–Nagaev estimator under two observation schemes. For the Studentized Lotka–Nagaev estimator based on one generation, the logarithmic relative error is $O(\left(1+x^{2+\rho }\right)n^{-\rho /2})$ for $0\leq x=o(\sqrt{n})$ and the Berry–Esseen bound is $O(n^{-\rho /2})$, with $p_{0}=0$ and a finite (2+ρ)th moment. For the self-normalized process based on centered generation increments divided by $\sqrt{X_{k}}$, the ratio to the corresponding normal tail tends to one uniformly for $0\leq x=o(n^{\rho /(4+2\rho )})$. In the Böttcher case $p_{0}=p_{1}=0$, Duan et al. [8], cf. Theorem 2 therein, obtained a logarithmic relative error of order $O(\left(1+x^{2+\rho }\right)k_{0}^{-n\rho /2})$ for the Studentized Lotka–Nagaev estimator on the range $0\leq x=o(k_{0}^{n/2})$, where $k_{0}=\min\left\{j\geq 2:p_{j}>0\right\}$. Their proof uses the total probability formula to condition on $X_{n}=k$, uses the independence of that generation’s offspring from $X_{n}$, and splits according to the empirical centering correction before applying the independent self-normalized estimate of Jing, Shao, and Wang [9]. For related results on the standardized Lotka–Nagaev estimator, we refer to [10,11,12]. General Cramér-type moderate deviation results for martingale are given in [13,14].

In this paper, we study deviation probabilities and self-normalized normal approximations for the Harris estimator, defined by

(3) $${\hat{m}}_{n}=\frac{\sum_{k=1}^{n}X_{k}}{\sum_{k=0}^{n-1}X_{k}}.$$

Unlike the Lotka–Nagaev estimator $X_{n+1}/X_{n}$, the Harris estimator pools the offspring observations over all generations up to time *n*. Bercu and Touati [15], cf. Corollary 5.3 therein, obtained exponential deviation inequalities for the Harris estimator with a local exponential-moment condition. In this paper, we obtain relative error bounds for the normal approximation with a finite (2+ρ)th moment assumption. Suppose that the individual offspring numbers are observed and that $E|X_{1}{-m|}^{2+\rho }<\infty$ for some 0<ρ≤1. Define the Studentized Harris Estimator by

(4) $$T_{n}^{H}=\frac{S_{n-1}\left({\hat{m}}_{n}-m\right)}{{\left(\sum_{k=1}^{n}\sum_{i=1}^{X_{k-1}}{\left(Y_{k,i}-{\hat{m}}_{n}\right)}^{2}\right)}^{1/2}}.$$

The main results give relative error bounds for the normal approximation to the Studentized Harris estimator under a finite (2+ρ)th moment assumption, both without extinction and conditional on survival. For the Harris estimator, earlier offspring numbers determine later generation sizes, and hence the number of subsequent observations in the pooled sample. Conditional on $S_{n-1}=t$, these observations need not have the law of *t* independent offspring variables. Conditioning on the entire sequence of generation sizes also constrains the offspring sums within each generation. Thus, the single-generation conditioning argument of Duan et al. [8] cannot be applied directly to the pooled Harris sample.

For $p_{0}=0$ and $p_{1}\in \left(0,1\right)$, the proof of Theorem 1 starts from the usual branching decomposition at an intermediate generation. Conditional on the observed history, the descendant subtrees are independent and the theorem of Jing, Shao, and Wang [9] applies to their centered totals. The normalizing factor is the square root of the sum of squared subtree totals. Each square contains cross terms, so this quadratic sum differs from the sum of squared individual offspring residuals used here.

The main step is to compare these two quadratic sums uniformly in subtree depth and to control the generations preceding the split. Lemma 1 gives uniform moment bounds and controls the deviations of both quadratic sums from their common conditional mean. Together with population growth and bounds for the early generations, this yields exceptional probabilities that remain exponentially small after division by the normal tail on $0\leq x\leq c\sqrt{n}$. The usual variance identity then gives the Studentized result. Thus, the independent self-normalized theorem is used in its existing form; the argument specific to the Harris estimator transfers the bound to the pooled sum and its normalizing factor. For a single generation, the independent variables are the offspring observations themselves, so this comparison of quadratic sums and the control of preceding generations are unnecessary.

For $p_{0}>0$, Theorem 2 uses a different argument. The Harris–Sevast’yanov transformation gives a lower-tail estimate for the population size conditional on survival. We use independence and moment estimates for the original descendant trees under P and control survival conditioning by a conditional mixture identity. A Berry–Esseen estimate for the subtree totals together with control of the normalizing factor gives a Berry–Esseen bound that decays exponentially fast with the generation *n*. Division by the normal tail then gives the stated moderate deviation range. The same estimates hold conditional on $X_{n}>0$. In both cases, $T_{n}^{H}$ is computed from the original tree.

In the Böttcher case $p_{0}=p_{1}=0$, the population has a deterministic exponential lower bound. Theorem 3 follows by using this bound in the proof of Theorem 1. Relative to Theorem 2 of Duan et al. [8], Theorem 3 establishes the normal-tail approximation for the Harris estimator, pooling all observed generations with its pooled empirical variance under the same Böttcher and moment assumptions. The additional argument handles the dependent observation count and the comparison of quadratic sums described above. The range $0\leq x\leq c\sqrt{n}$ obtained here is narrower than their single-generation range; a sharp range for the Harris estimator in this case is not claimed. Together, the three theorems cover all nondegenerate supercritical offspring laws satisfying the stated moment condition.

The paper is organized as follow: Section 2 states the theorems and their consequences; Section 3 gives an application to the construction of confidence-interval; Section 4 examines its numerical behavior; Section 5 contains the proofs; and Section 6 discusses the scope and limitations of the results.

## 2. Main Results

Set

(5) $$M_{n}^{H}=\sum_{k=1}^{n}\sum_{i=1}^{X_{k-1}}\left(Y_{k,i}-m\right),\;V_{n}^{2}=\sum_{k=1}^{n}\sum_{i=1}^{X_{k-1}}{\left(Y_{k,i}-m\right)}^{2}.$$

Then, $M_{n}^{H}=S_{n-1}\left({\hat{m}}_{n}-m\right)$, where $S_{n-1}=\sum_{k=0}^{n-1}X_{k}$. Define

(6) $$W_{n}^{H}=\frac{M_{n}^{H}}{V_{n}},\;{\hat{V}}_{n}^{2}=\sum_{k=1}^{n}\sum_{i=1}^{X_{k-1}}{\left(Y_{k,i}-{\hat{m}}_{n}\right)}^{2},\;T_{n}^{H}=\frac{M_{n}^{H}}{{\hat{V}}_{n}}.$$

Notice that

(7) $${\hat{V}}_{n}^{2}=V_{n}^{2}-\frac{{\left(M_{n}^{H}\right)}^{2}}{S_{n-1}}.$$

Let Φ denote the standard normal distribution function. The following theorem gives self-normalized Cramér-type moderate deviations for the Studentized Harris estimator.

**Theorem** **1.** *Assume that $p_{0}=0$, $p_{1}\in \left(0,1\right)$, $m=EX_{1}<\infty$, and $0<\sigma^{2}=\mathrm{Var}\left(X_{1}\right)<\infty$. Let ρ∈(0,1] and suppose that $E|X_{1}{-m|}^{2+\rho }<\infty$. Then, there exists a constant c>0 such that*

(8) $$\underset{0\leq x\leq c\sqrt{n}}{\sup}\left|\log\frac{P(T_{n}^{H}\geq x)}{1-\Phi (x)}\right|=O\left(e^{-cn}\right),\;n\to \infty .$$

*Furthermore, the equality in (8) remains valid when $\frac{P(T_{n}^{H}\geq x)}{1-\Phi (x)}$ is replaced by $\frac{P(T_{n}^{H}\leq -x)}{\Phi (-x)}$.*

Theorem 1 yields the following moderate deviation principle.

**Corollary** **1.** *Assume that the conditions of Theorem 1 are satisfied. Let ${\left(a_{n}\right)}_{n\geq 1}$ be any sequence of positive real numbers satisfying $a_{n}\to \infty$ and $a_{n}=o\left(\sqrt{n}\right)$. Then, $T_{n}^{H}/a_{n}$ satisfies a moderate deviation principle with speed $a_{n}^{2}$ and rate function $I\left(x\right)=x^{2}/2$. More precisely, for every Borel set B⊂R, we have*

(9) $$\begin{array}{cc} -\underset{x\in B^{\circ }}{\inf}I\left(x\right) & \leq \underset{n\to \infty }{\lim\;\inf}\frac{1}{a_{n}^{2}}\log P\left(T_{n}^{H}/a_{n}\in B\right)\\ \; & \leq \underset{n\to \infty }{\lim\;\sup}\frac{1}{a_{n}^{2}}\log P\left(T_{n}^{H}/a_{n}\in B\right)\leq -\underset{x\in \bar{B}}{\inf}I\left(x\right), \end{array}$$

*where $B^{\circ }$ and $\bar{B}$ denote the interior and the closure of B, respectively.*

Theorem 1 also implies the following Berry–Esseen bound.

**Corollary** **2.** *Assume that the conditions of Theorem 1 are satisfied. Then, there exists a constant c>0 such that*

(10) $$\underset{x\in R}{\sup}\left|P\left(T_{n}^{H}\leq x\right)-\Phi \left(x\right)\right|=O\left(e^{-cn}\right),\;n\to \infty .$$

For the case $p_{0}>0$, write

(11) $$f\left(s\right)=Es^{X_{1}},\;S=\underset{k\geq 0}{⋂}\left\{X_{k}>0\right\},\;q=P\left(S^{c}\right).$$

With m>1, q∈(0,1) is the extinction probability and satisfies f(q)=q. For an event *B*, put

$$P_{S}\left(B\right)=P\left(B|S\right),\;P_{n}^{+}\left(B\right)=P\left(B|X_{n}>0\right).$$

Write $E_{S}$ for the expectation under $P_{S}$. Under both conditional laws, $T_{n}^{H}$ is computed from the offspring observations in the original tree.

For 0<ρ≤1, set

(12) $$d=f^{\prime }\left(q\right),\;\alpha =\frac{1}{2}\log\frac{2}{1+d},\;\gamma_{S}=\frac{\rho \alpha }{4(4+\rho )},$$

where 0<d<1.

**Theorem** **2.** *Assume that $p_{0}>0$, $1<m=EX_{1}<\infty$, and $0<\sigma^{2}=\mathrm{Var}\left(X_{1}\right)<\infty$. Let ρ∈(0,1] and suppose that $E|X_{1}{-m|}^{2+\rho }<\infty$. Then, there exists a constant c>0 such that*

(13) $$\underset{0\leq x\leq c\sqrt{n}}{\sup}\left|\log\frac{P_{S}\left(T_{n}^{H}\geq x\right)}{1-\Phi (x)}\right|=O\left(e^{-cn}\right),\;n\to \infty .$$

*Furthermore, (13) remains valid when the ratio $\frac{P_{S}\left(T_{n}^{H}\geq x\right)}{1-\Phi (x)}$ is replaced by $\frac{P_{S}\left(T_{n}^{H}\leq -x\right)}{\Phi (-x)}$. Moreover,*

(14) $$\underset{z\in R}{\sup}\left|P_{S}\left(T_{n}^{H}\leq z\right)-\Phi \left(z\right)\right|=O\left(e^{-\gamma_{S}n}\right),\;n\to \infty ,$$

*where $\gamma_{S}$ is defined in (12). All these estimates remain valid with $P_{S}$ replaced by $P_{n}^{+}$.*

Theorem 2 also gives a conditional moderate deviation principle.

**Corollary** **3.** *Assume that the conditions of Theorem 2 are satisfied. Let $\left(a_{n}\right)$ be positive with $a_{n}\to \infty$ and $a_{n}=o\left(\sqrt{n}\right)$, and let $Q_{n}$ denote either $P_{S}$ or $P_{n}^{+}$. Then, for every Borel set B⊂R,*

(15) $$\begin{array}{cc} -\underset{x\in B^{\circ }}{\inf}I\left(x\right) & \leq \underset{n\to \infty }{\lim\;\inf}\frac{1}{a_{n}^{2}}\log Q_{n}\left(T_{n}^{H}/a_{n}\in B\right)\\ \; & \leq \underset{n\to \infty }{\lim\;\sup}\frac{1}{a_{n}^{2}}\log Q_{n}\left(T_{n}^{H}/a_{n}\in B\right)\leq -\underset{x\in \bar{B}}{\inf}I\left(x\right), \end{array}$$

*where $I\left(x\right)=x^{2}/2$.*

We next consider the Böttcher case $p_{0}=p_{1}=0$. Put

(16) $$k_{0}=\min\left\{j\geq 2:p_{j}>0\right\}.$$

Every individual has at least $k_{0}$ offspring, so $X_{t}\geq k_{0}^{t}$ a.s. This deterministic bound replaces the population lower-tail estimate in the proof of Theorem 1.

**Theorem** **3.** *Assume that $p_{0}=p_{1}=0$, $m=EX_{1}<\infty$, and $0<\sigma^{2}=\mathrm{Var}\left(X_{1}\right)<\infty$. Let ρ∈(0,1] and suppose that $E|X_{1}{-m|}^{2+\rho }<\infty$. Then, there exists a constant c>0 such that*

(17) $$\underset{0\leq x\leq c\sqrt{n}}{\sup}\left|\log\frac{P(T_{n}^{H}\geq x)}{1-\Phi (x)}\right|=O\left(e^{-cn}\right),\;n\to \infty .$$

*Furthermore, the equality in (17) remains valid when $\frac{P(T_{n}^{H}\geq x)}{1-\Phi (x)}$ is replaced by $\frac{P(T_{n}^{H}\leq -x)}{\Phi (-x)}$.*

Theorem 3 yields the following two consequences.

**Corollary** **4.** *Assume that the conditions of Theorem 3 are satisfied. Let $\left(a_{n}\right)$ be positive with $a_{n}\to \infty$ and $a_{n}=o\left(\sqrt{n}\right)$. Then, $T_{n}^{H}/a_{n}$ satisfies a moderate deviation principle with speed $a_{n}^{2}$ and rate function $I\left(x\right)=x^{2}/2$. More precisely, for every Borel set B⊂R, it holds that*

(18) $$\begin{array}{cc} -\underset{x\in B^{\circ }}{\inf}I\left(x\right) & \leq \underset{n\to \infty }{\lim\;\inf}\frac{1}{a_{n}^{2}}\log P\left(T_{n}^{H}/a_{n}\in B\right)\\ \; & \leq \underset{n\to \infty }{\lim\;\sup}\frac{1}{a_{n}^{2}}\log P\left(T_{n}^{H}/a_{n}\in B\right)\leq -\underset{x\in \bar{B}}{\inf}I\left(x\right). \end{array}$$

**Corollary** **5.** *Assume that the conditions of Theorem 3 are satisfied. Then, there exists a constant c>0 such that*

(19) $$\underset{z\in R}{\sup}\left|P\left(T_{n}^{H}\leq z\right)-\Phi \left(z\right)\right|=O\left(e^{-cn}\right),\;n\to \infty .$$

## 3. Asymptotic Confidence Intervals

Assume that the offspring numbers ${\left(Y_{k,i}\right)}_{1\leq k\leq n,\;1\leq i\leq X_{k-1}}$ can be observed for some *n*. The following result gives an asymptotic confidence interval for *m*.

**Corollary** **6.** *Assume the conditions of one of Theorems 1–3. Under the conditions of Theorem 1 or 3, set $Q_{n}=P$. Under the conditions of Theorem 2, let $Q_{n}$ be either $P_{S}$ or $P_{n}^{+}$. Let $\kappa_{n}\in \left(0,1\right)$ satisfy*

(20) $$|\log\kappa_{n}|=o\left(n\right),\;n\to \infty$$

*and set*

(21) $$\Delta_{n}=\frac{\Phi^{-1}\left(1-\kappa_{n}/2\right)}{S_{n-1}}{\left\{\sum_{k=1}^{n}\sum_{i=1}^{X_{k-1}}{\left(Y_{k,i}-{\hat{m}}_{n}\right)}^{2}\right\}}^{1/2}.$$

*Then, $\left[A_{n},B_{n}\right]$ is an asymptotic $1-\kappa_{n}$ confidence interval for m under $Q_{n}$, where*

(22) $$A_{n}={\hat{m}}_{n}-\Delta_{n},\;B_{n}={\hat{m}}_{n}+\Delta_{n}.$$

*In fact, for some c>0,*

(23) $$Q_{n}\left\{m\notin \left[A_{n},B_{n}\right]\right\}=\kappa_{n}\left\{1+O\left(e^{-cn}\right)\right\}.$$

**Proof.**  Set $z_{n}=\Phi^{-1}\left(1-\kappa_{n}/2\right)$. The standard normal tail bounds give

$$\frac{1}{\sqrt{2\pi }\left(1+x\right)}e^{-x^{2}/2}\leq 1-\Phi \left(x\right)\leq \frac{1}{\sqrt{\pi }\left(1+x\right)}e^{-x^{2}/2},\;x>0.$$

For $z_{n}\geq 1$, it follows that

$$\frac{\kappa_{n}}{2}=1-\Phi \left(z_{n}\right)\leq \frac{1}{\sqrt{\pi }}e^{-z_{n}^{2}/2}.$$

Taking the logarithms gives

$$z_{n}^{2}\leq 2\left|\log\kappa_{n}\right|+C.$$

Thus, it holds that

$$0\leq z_{n}\leq C\left(1+\sqrt{|\log\kappa_{n}|}\right).$$

It follows from (20) that $z_{n}=o\left(\sqrt{n}\right)$. Hence, $z_{n}+1\leq c\sqrt{n}$ for all sufficiently large *n*, where c>0 is sufficiently small. Theorems 1–3 give the same tail estimate under the corresponding measure $Q_{n}$. By continuity from below, applying this estimate for $z_{n}<y\leq z_{n}+1$ and letting $y↓z_{n}$ gives the corresponding estimates for $Q_{n}\left(T_{n}^{H}>z_{n}\right)$ and $Q_{n}\left(T_{n}^{H}<-z_{n}\right)$. Consequently, we get

$$\begin{array}{cc} Q_{n}\left\{T_{n}^{H}>z_{n}\right\} & =\frac{\kappa_{n}}{2}\left\{1+O\left(e^{-cn}\right)\right\},\\ Q_{n}\left\{T_{n}^{H}<-z_{n}\right\} & =\frac{\kappa_{n}}{2}\left\{1+O\left(e^{-cn}\right)\right\}. \end{array}$$

By (6), (21), and (22), we have

$$\begin{array}{cc} \left\{T_{n}^{H}\leq z_{n}\right\} & =\{m\geq A_{n}\},\\ \left\{T_{n}^{H}\geq -z_{n}\right\} & =\{m\leq B_{n}\}. \end{array}$$

Consequently, it holds that

$$\begin{array}{cc} \left\{m\notin \left[A_{n},B_{n}\right]\right\} & =\left\{m<A_{n}\right\}\cup \left\{m>B_{n}\right\}\\ \; & =\left\{T_{n}^{H}>z_{n}\right\}\cup \left\{T_{n}^{H}<-z_{n}\right\}, \end{array}$$

where the two events in the last union are disjoint. Therefore, it holds that

$$Q_{n}\left\{m\notin \left[A_{n},B_{n}\right]\right\}=\kappa_{n}\left\{1+O\left(e^{-cn}\right)\right\},$$

which gives (23). Thus, $\left[A_{n},B_{n}\right]$ is an asymptotic $1-\kappa_{n}$ confidence interval for *m*. □

**Remark** **1.** *The Harris estimator is determined by $X_{0},\ldots ,X_{n}$. To compute ${\hat{V}}_{n}$, it suffices to additionally record the sum of squared offspring numbers:*

(24) $$Q_{n}=\sum_{k=1}^{n}\sum_{i=1}^{X_{k-1}}Y_{k,i}^{2},\;{\hat{V}}_{n}^{2}=Q_{n}-\frac{{\left(\sum_{k=1}^{n}X_{k}\right)}^{2}}{S_{n-1}}.$$

*Individual identities need not be retained. Generation sizes alone do not determine $Q_{n}$, as the offspring numbers (1,3) and (2,2) have the same sum but different sums of squares.*

**Remark** **2.** *Let $z_{n}=\Phi^{-1}\left(1-\kappa_{n}/2\right)$ and ${\hat{\sigma }}_{n}^{2}={\hat{V}}_{n}^{2}/S_{n-1}$. The interval $\left[A_{n},B_{n}\right]$ is the usual Wald interval ${\hat{m}}_{n}\pm z_{n}{\hat{\sigma }}_{n}/\sqrt{S_{n-1}}$. Corollary 6 bounds the relative error in the noncoverage probability when $|\log\kappa_{n}|=o\left(n\right)$, including fixed confidence levels. Under the conditions of Theorem 2, coverage is conditional on S or $X_{n}>0$. The constants depend on the offspring law. The result is asymptotic, and does not provide a computable finite-sample coverage guarantee.*

## 4. Numerical Study

We assess the finite-generation performance of the Studentized Harris estimator and the confidence intervals in Corollary 6. The simulations examine coverage, interval length, and the relative accuracy of the normal-tail approximation in Theorem 1. The offspring laws span bounded, geometric, and heavy-tailed distributions. Additional comparisons examine slow population growth and variation in distributional shape at a common mean and variance. Throughout, $p_{0}=0$ and $p_{1}\in \left(0,1\right)$.

### 4.1. Offspring Laws and Simulation Design

Models B1 and B2 place probabilities (0.5,0.5) and (0.4,0.6), respectively, on {1,2}; B3 and B4 place probabilities (0.3,0.3,0.4) and (0.2,0.5,0.3) on {1,2,3}. These four bounded laws serve as baselines. Model H has $p_{1}=0.95$ and $p_{11}=0.05$, whereas R has

$$p_{1}=0.55,\;p_{101}=\frac{0.05}{99},\;p_{2}=0.45-\frac{0.05}{99}.$$

Both have mean 1.5. Model H allows prolonged single-offspring reproduction, while R introduces rare extreme offspring counts. Model G is geometric, with $p_{j}=\left(2/3\right){\left(1/3\right)}^{j-1}$ for j≥1.

For the heavy-tailed model P, set Y=1+JK, where a=2.6, *K* is positive integer-valued with $P\left(K\geq k\right)=k^{-a}$, k≥1, and *J* is an independent Bernoulli variable with $P\left(J=1\right)={\left[2\zeta \left(a\right)\right]}^{-1}$. Here, ζ denotes the Riemann zeta function. Then,

$$EY=1.5,\;\mathrm{Var}\left(Y\right)=\frac{2\zeta (a-1)-\zeta (a)}{2\zeta (a)}-\frac{1}{4}.$$

This law has a finite moment of order 2.5 and an infinite third moment; therefore, it satisfies the moment assumption of Theorem 1 with ρ=0.5. To examine sensitivity while holding the first two moments fixed, we also take $p_{1}=u$, u∈{0.68,0.74,0.80}, and set

$$p_{11}=\frac{2.75-2u}{72},\;p_{3}=u-0.5-9p_{11},\;p_{2}=1-u-p_{3}-p_{11}.$$

The resulting laws, denoted by S68, S74, and S80, have m=1.5 and $\sigma^{2}=2$ but differ in their higher moments. Table 1 summarizes the eleven laws.

For each law, we generate 200,000 independent trees from a single ancestor. For B1, H, R, G, P, and the sensitivity family, we record results at n=10,30,60; the supplementary comparisons for B2–B4 use n=10,30. Models use independent random-number streams. Within each model, observations at different generations and comparisons between interval constructions use the same trees. We report results for nominal 95% intervals; the corresponding 90% results are included in the supplementary output.

Finite-support laws are simulated using conditional multinomial counts. For G and P, counts for an initial set of offspring values are combined with conditional sampling of every remaining tail observation. This construction preserves the unbounded offspring laws. Every tree contributes to the reported estimates. The Supplementary Material provides the code, numerical output, and independent checks against individual-offspring simulation.

We consider the Studentized interval

$$\left[{\hat{m}}_{n}-z_{1-\kappa /2}\frac{{\hat{V}}_{n}}{S_{n-1}},\;{\hat{m}}_{n}+z_{1-\kappa /2}\frac{{\hat{V}}_{n}}{S_{n-1}}\right],\;z_{v}=\Phi^{-1}\left(v\right).$$

We quantify Monte Carlo uncertainty using pointwise 95% Wilson intervals for probability estimates and standard errors for mean interval lengths.

### 4.2. Coverage and Interval Length

Table 2 and Figure 1 report the results for B1, R, G, and P. At n=10, coverage ranges from 0.8205 for R to 0.934 for B1. At n=30, the range narrows to 0.9443–0.9502; at n=60, all four estimates lie between 0.9500 and 0.9508. Over the same range of generations, mean interval length decreases by more than three orders of magnitude in each model; thus, the gain in coverage is accompanied by a substantial increase in precision.

We compare these intervals with the known-variance benchmark ${\hat{m}}_{n}\pm z_{1-\kappa /2}\sigma /\sqrt{S_{n-1}}$, computed on the same trees. Table 3 gives the two coverage estimates at n=60, their paired difference, and its Monte Carlo standard error. For B1, R, G, P, and the sensitivity family, the two estimates differ by at most $10^{-4}$.

Coverage improves more gradually under H, rising from 0.3865 at n=10 to 0.7374 at n=30 and 0.8963 at n=60. The high probability of single-offspring reproduction delays branching along many paths. This is consistent with the slower improvement in coverage and the larger mean interval lengths shown in the supplementary H figure. Since H has the same offspring mean as B1, R, G, and P, this comparison illustrates the role of growth along individual trees.

The sensitivity family holds the first two moments fixed while varying the higher moments. As shown in Figure 2, coverage for S68, S74, and S80 approaches the nominal level, reaching 0.9501, 0.9497, and 0.9493, respectively, at n=60. Mean interval lengths nevertheless remain distinct: at n=60, the mean length for S80 is approximately 2.7 times that for S68. Thus, the comparison exhibits variation in interval precision among offspring laws with identical first two moments.

### 4.3. Relative Tail Accuracy

We estimate

$$\frac{P(T_{n}^{H}\geq x)}{1-\Phi (x)}\;\mathrm{and}\;\frac{P(T_{n}^{H}\leq -x)}{\Phi (-x)}$$

at 31 equally spaced points in [0.1,2.5]. Figure 3 and Figure 4 display the estimates at n=10,30,60, with 1 as the normal reference. Shaded bands are obtained by dividing the pointwise 95% Wilson intervals for the empirical probabilities by the corresponding normal tail probabilities. Panels share a common scale within each figure; the left-tail figure uses a logarithmic vertical axis.

At n=10, the right-tail ratios lie below 1 and the left-tail ratios above 1, with larger discrepancies toward the upper end of the grid. This asymmetry diminishes as *n* increases. B1 is already close to the normal reference at n=30, whereas R and P improve more gradually. At that generation and x=2.5, their right-tail ratios are 0.504 and 0.658 and their left-tail ratios are 2.306 and 1.464, respectively. By n=60, all four models track the normal reference closely in both tails over the plotted range.

Table 4 gives the endpoint estimates and their uncertainty intervals. At n=60, the eight ratios lie between 0.974 and 1.018, and all eight Wilson intervals contain 1. For P, for which the third moment is infinite, the right- and left-tail ratios are 0.993 and 0.992. Thus, the agreement at the largest recorded generation extends to the heavy-tailed example.

For the principal bounded and unbounded offspring laws, the results at n=60 combine near-nominal coverage, substantially shorter intervals, and close agreement with the normal approximation in both tails. The comparisons at n=10 and n=30 document the development of this accuracy as the population grows. The additional experiments with H and the sensitivity family show how population growth and offspring distributional shape influence the finite-generation performance of the procedure.

## 5. Proofs of the Main Results

Throughout this section, C,c>0 may vary from line to line and depend only on ρ and the offspring law.

### 5.1. Auxiliary Lemmas

For the process initiated by one individual, let $M_{r}^{H}$ and $V_{r}$ be defined by (5) with *n* replaced by *r*.

**Lemma** **1.** *Assume m>1, $0<\sigma^{2}<\infty$ and $E|X_{1}{-m|}^{2+\rho }<\infty$ for some 0<ρ≤1. Then, for every r≥1, we have*

(25) $$\begin{array}{cc} \; & EM_{r}^{H}=0,\;E{\left(M_{r}^{H}\right)}^{2}=EV_{r}^{2}=\sigma^{2}\frac{m^{r}-1}{m-1}, \end{array}$$

(26) $$\begin{array}{cc} \; & E|M_{r}^{H}{|}^{2+\rho }+EV_{r}^{2+\rho }\leq C{\left(\sigma^{2}\frac{m^{r}-1}{m-1}\right)}^{1+\rho /2}. \end{array}$$

*Let ${\left(M_{r}^{H,(j)},V_{r}^{\left(j\right)}\right)}_{j\geq 1}$ be independent copies of $\left(M_{r}^{H},V_{r}\right)$. Then, for all N,r≥1 and 0<η≤1/2,*

(27) $$\begin{array}{cc} \; & P(\left\{\left|\sum_{j=1}^{N}{\left(M_{r}^{H,(j)}\right)}^{2}-NEV_{r}^{2}\right|>\eta NEV_{r}^{2}\right\}\\ \; & \;\cup \left\{\left|\sum_{j=1}^{N}{\left(V_{r}^{\left(j\right)}\right)}^{2}-NEV_{r}^{2}\right|>\eta NEV_{r}^{2}\right\})\leq C\eta^{-(1+\rho /2)}N^{-\rho /2}, \end{array}$$

*where C is independent of N and r.*

**Proof.**  Put ν=1+ρ/2. Since 1<ν≤3/2, the conditional von Bahr–Esseen inequality [16] gives

$$E\left(|X_{k+1}-mX_{k}{|}^{\nu }|F_{k}\right)\leq CX_{k},\;{∥X_{k+1}-mX_{k}∥}_{\nu }\leq C{\left(EX_{k}\right)}^{1/\nu }=Cm^{k/\nu }.$$

Since $X_{k+1}=mX_{k}+\left(X_{k+1}-mX_{k}\right)$, iterating Minkowski’s inequality yields

(28) $$∥X_{k}{∥}_{\nu }\leq m^{k}+C\sum_{j=0}^{k-1}m^{k-1-j+j/\nu }\leq Cm^{k},\;k\geq 0.$$

The increments of $M_{r}^{H}$ are $X_{k}-mX_{k-1}$, and

$$E\left\{{\left(X_{k}-mX_{k-1}\right)}^{2}|F_{k-1}\right\}=\sigma^{2}X_{k-1}.$$

Therefore, orthogonality gives

$$EM_{r}^{H}=0,\;E{\left(M_{r}^{H}\right)}^{2}=\sigma^{2}\sum_{k=0}^{r-1}m^{k}=EV_{r}^{2}.$$

To estimate $V_{r}$, condition on $F_{k-1}$ to obtain

$$E\left[{\left\{\sum_{i=1}^{X_{k-1}}{\left(Y_{k,i}-m\right)}^{2}\right\}}^{\nu }\;|\;F_{k-1}\right]\leq X_{k-1}^{\nu }E{\left|X_{1}-m\right|}^{2\nu }.$$

Consequently,

$${∥\sum_{i=1}^{X_{k-1}}{\left(Y_{k,i}-m\right)}^{2}∥}_{\nu }\leq C{∥X_{k-1}∥}_{\nu }\leq Cm^{k-1}.$$

By (28) and Minkowski’s inequality, we get

$$∥V_{r}^{2}{∥}_{\nu }\leq C\sum_{k=1}^{r}m^{k-1}\leq Cm^{r}.$$

This proves the bound for $EV_{r}^{2+\rho }$.

Conditionally on $F_{k-1}$, Rosenthal’s inequality [17] gives

$$E\{|X_{k}-mX_{k-1}{|}^{2+\rho }|F_{k-1}\}\leq C\left\{X_{k-1}E{\left|X_{1}-m\right|}^{2+\rho }+{\left(\sigma^{2}X_{k-1}\right)}^{1+\rho /2}\right\}.$$

By (28),

$$E|X_{k}-mX_{k-1}{|}^{2+\rho }\leq C\left\{m^{k-1}+EX_{k-1}^{\nu }\right\}\leq Cm^{\nu (k-1)}.$$

Burkholder’s inequality [18] and Minkowski’s inequality in $L^{\nu }$ now imply

$$\begin{array}{cc} E|M_{r}^{H}{|}^{2+\rho } & \leq CE{\left\{\sum_{k=1}^{r}{\left(X_{k}-mX_{k-1}\right)}^{2}\right\}}^{\nu }\\ \; & \leq C{\left\{\sum_{k=1}^{r}{\left(E|X_{k}-mX_{k-1}{|}^{2+\rho }\right)}^{1/\nu }\right\}}^{\nu }\leq Cm^{\nu r}. \end{array}$$

Since $\sigma^{2}\left(m^{r}-1\right)/\left(m-1\right)≍m^{r}$, this proves (26).

Finally,

$$E{\left|{\left(M_{r}^{H}\right)}^{2}-E{\left(M_{r}^{H}\right)}^{2}\right|}^{1+\rho /2}+E{\left|V_{r}^{2}-EV_{r}^{2}\right|}^{1+\rho /2}\leq C{\left(EV_{r}^{2}\right)}^{1+\rho /2}.$$

Apply the von Bahr–Esseen inequality separately to the sums of the centered variables ${\left(M_{r}^{H,(j)}\right)}^{2}-E{\left(M_{r}^{H}\right)}^{2}$ and ${\left(V_{r}^{\left(j\right)}\right)}^{2}-EV_{r}^{2}$. Markov’s inequality at level $\eta NEV_{r}^{2}$ then yields (27). No part of the argument requires $p_{0}=0$ or $p_{1}>0$. □

**Lemma** **2** (Jing, Shao, and Wang [9]). *Let 0<ρ≤1, and let ${\left(Z_{j}\right)}_{j\geq 1}$ be i.i.d. copies of a centered random variable Z such that*

$$EZ^{2}=\tau^{2}>0,\;E{\left|Z\right|}^{2+\rho }\leq C_{0}\tau^{2+\rho },$$

*where $C_{0}\geq 1$. Denote*

$$S_{N}=\sum_{j=1}^{N}Z_{j},\;V_{N}^{2}=\sum_{j=1}^{N}Z_{j}^{2}.$$

*Then, there exist constants C>0 and $c_{1}>0$ and an integer $N_{0}\geq 1$, depending only on $C_{0}$ and ρ such that for every $N\geq N_{0}$ and $0\leq x\leq c_{1}\sqrt{N}$,*

(29) $$\left|\log\frac{P(S_{N}\geq xV_{N})}{1-\Phi (x)}\right|\leq C\frac{1+x^{2+\rho }}{N^{\rho /2}}.$$

**Proof.**  The stated uniform bound follows from Theorem 2.1 of Jing, Shao, and Wang [9]. In the i.i.d. setting, their error quantity reduces to

$$\begin{array}{cc} \Delta_{N,x}= & \frac{{\left(1+x\right)}^{2}}{\tau^{2}}E\left[Z^{2}1_{\left\{|Z|>\sqrt{N}\tau /\left(1+x\right)\right\}}\right]\\ \; & +\frac{{\left(1+x\right)}^{3}}{\sqrt{N}\tau^{3}}E\left[{\left|Z\right|}^{3}1_{\left\{|Z|\leq \sqrt{N}\tau /\left(1+x\right)\right\}}\right]. \end{array}$$

The moment assumption gives

$$\begin{array}{cc} E\left[Z^{2}1_{\left\{|Z|>a\right\}}\right] & \leq a^{-\rho }E{\left|Z\right|}^{2+\rho },\\ E\left[{\left|Z\right|}^{3}1_{\left\{|Z|\leq a\right\}}\right] & \leq a^{1-\rho }E{\left|Z\right|}^{2+\rho }. \end{array}$$

Therefore, taking $a=\sqrt{N}\tau /\left(1+x\right)$ yields

(30) $$\Delta_{N,x}\leq CC_{0}\frac{{\left(1+x\right)}^{2+\rho }}{N^{\rho /2}}\leq CC_{0}\frac{1+x^{2+\rho }}{N^{\rho /2}}.$$

The exponential estimate in Theorem 2.1 applies whenever $x^{2}\tau^{2}\leq N\tau^{2}$ and $\Delta_{N,x}\leq {\left(1+x\right)}^{2}/A_{\mathrm{JSW}}$, where $A_{\mathrm{JSW}}>0$ is an absolute constant. By (30), the latter condition is ensured by

$$CC_{0}{\left(\frac{1+x}{\sqrt{N}}\right)}^{\rho }\leq \frac{1}{A_{\mathrm{JSW}}}.$$

Choose $c_{1}\in \left(0,1\right)$ sufficiently small and then choose $N_{0}$ sufficiently large. For every $N\geq N_{0}$ and $0\leq x\leq c_{1}\sqrt{N}$, both conditions are satisfied. Theorem 2.1 of Jing, Shao, and Wang [9] then yields (29), with constants depending only on $C_{0}$ and ρ. □

**Lemma** **3.** *Assume $p_{0}=0$, $p_{1}\in \left(0,1\right)$ and m<∞. Set $\theta =(1+p_{1})/2$ and $a_{0}=c_{0}=-\frac{1}{2}\log\theta >0$. Then,*

(31) $$P\left(X_{t}<e^{a_{0}t}\right)\leq e^{-c_{0}t},\;t\in N.$$

**Proof.**  For positive numbers $y_{1},\ldots ,y_{k}$, the arithmetic–harmonic mean inequality gives

$$\frac{k}{y_{1}+\ldots +y_{k}}\leq \frac{1}{k}\sum_{i=1}^{k}\frac{1}{y_{i}}.$$

For independent copies $Y_{i}$ of $X_{1}$, it follows that

$$kE{\left(\sum_{i=1}^{k}Y_{i}\right)}^{-1}\leq E\left(Y_{1}^{-1}\right)\leq p_{1}+\frac{1-p_{1}}{2}=\theta <1.$$

Therefore, the branching property yields

$$E\left(X_{t+1}^{-1}|F_{t}\right)\leq \theta X_{t}^{-1},\;EX_{t}^{-1}\leq \theta^{t}.$$

Markov’s inequality gives

$$P\left(X_{t}<e^{a_{0}t}\right)\leq e^{a_{0}t}EX_{t}^{-1}\leq e^{a_{0}t}\theta^{t}\leq e^{-a_{0}t},$$

which proves the assertion. The exact reciprocal contraction factor is $\lambda =E(Y_{1}^{-1})$, that is, the displayed bound is attained at k=1 when taking the supremum over *k*. Jensen’s inequality also gives $m^{-1}\leq \lambda \leq \theta$; hence, $\log m\geq 2a_{0}$. □

**Lemma** **4.** *Assume $p_{0}>0$ and 1<m<∞. With the notation in (11) and (12),*

(32) $$P_{S}\left(X_{t}<e^{\alpha t}\right)\leq e^{-\alpha t},\;t=0,1,\ldots .$$

*For every event B and integer ℓ≥0,*

(33) $$\left|P_{S}\left(B|F_{\ell }\right)-P\left(B|F_{\ell }\right)\right|\leq q^{X_{\ell }},\;P_{S}\mathrm{-a}.s.$$

**Proof.**  The Harris–Sevast’yanov transformation has generating function

(34) $$g\left(s\right)=\frac{f(q+(1-q)s)-q}{1-q},\;0\leq s\leq 1;$$

see Schuh [19]. We verify the marginal identity needed here. Let $K_{t}$ count the individuals in generation *t*, whose descendants survive forever. Under P, the corresponding indicators, conditional on $F_{t}$, are independent with success probability 1−q. Since $\left\{K_{t}=0\right\}=S^{c}$ a.s.,

(35) $$E_{S}s^{K_{t}}=\frac{f^{\circ t}\left(q+\left(1-q\right)s\right)-q}{1-q}=g^{\circ t}\left(s\right).$$

Thus, $K_{t}$ under $P_{S}$ has the same distribution as the generation size of a Galton–Watson process with generating function *g* and one ancestor.

We have g(0)=0, $g^{\prime }\left(1\right)=m$, and $g^{\prime }\left(0\right)=f^{\prime }\left(q\right)=d$. Strict convexity of *f*, together with f(q)=q and f(1)=1, gives 0<d<1. Applying Lemma 3 to the process with generating function *g* and using $K_{t}\leq X_{t}$ proves (32).

The branching property gives $P\left(S^{c}|F_{\ell }\right)=q^{X_{\ell }}$. On $\left\{X_{\ell }>0\right\}$, the conditional law of total probability gives

$$\begin{array}{cc} P(B|F_{\ell })= & \left(1-q^{X_{\ell }}\right)P_{S}\left(B|F_{\ell }\right)\\ \; & +q^{X_{\ell }}P\left(B|F_{\ell },S^{c}\right). \end{array}$$

Subtracting $P_{S}\left(B|F_{\ell }\right)$ proves (33). □

**Lemma** **5.** *Let $Z_{1},\ldots ,Z_{N}$ be i.i.d. centered random variables with $EZ_{1}^{2}=\tau^{2}>0$ and $E|Z_{1}{|}^{2+\rho }\leq K\tau^{2+\rho }$, where 0<ρ≤1. Then,*

(36) $$\underset{z\in R}{\sup}\left|P\left(\frac{\sum_{j=1}^{N}Z_{j}}{\tau \sqrt{N}}\leq z\right)-\Phi \left(z\right)\right|\leq C_{\rho }KN^{-\rho /2}.$$

**Proof.**  See, for instance, Chen and Shao [20] for a proof of this Berry–Esseen bound. □

### 5.2. Proof of Theorem 1

**Proof.**  Put

$$\ell_{n}=\left\lfloor n/2\right\rfloor ,\;r_{n}=n-\ell_{n}.$$

With $a_{0}=c_{0}$ as in Lemma 3, put

(37) $$\gamma =\frac{\rho a_{0}}{12(2+\rho )}<\min\left\{\frac{\log m}{8},\frac{\rho a_{0}}{6(2+\rho )}\right\},\;\eta_{n}=e^{-\gamma n}.$$

The first inequality follows from $\log m\geq 2a_{0}$. Set $\beta_{0}=\rho a_{0}/8$ and $\varepsilon_{0}=\sqrt{\beta_{0}/16}$.

For $1\leq j\leq X_{\ell_{n}}$, let $M_{r_{n}}^{H,(j)}$ and $V_{r_{n}}^{\left(j\right)}$ be the quantities in (5) computed from the descendants of the *j*th individual over the next $r_{n}$ generations. Conditional on $F_{\ell_{n}}$, these pairs are independent and each has the same distribution as $\left(M_{r_{n}}^{H},V_{r_{n}}\right)$. Hence, by the branching property,

(38) $$M_{n}^{H}=M_{\ell_{n}}^{H}+\sum_{j=1}^{X_{\ell_{n}}}M_{r_{n}}^{H,(j)},\;V_{n}^{2}=V_{\ell_{n}}^{2}+\sum_{j=1}^{X_{\ell_{n}}}{\left(V_{r_{n}}^{\left(j\right)}\right)}^{2}.$$

Lemma 3 gives

(39) $$\begin{array}{cc} P(X_{\ell_{n}}<e^{a_{0}\ell_{n}}) & \leq e^{-c_{0}\ell_{n}}\leq Ce^{-cn}. \end{array}$$

Since $p_{0}=0$, $X_{\ell_{n}}\geq X_{k-1}\geq 1$ for $1\leq k\leq \ell_{n}$. Therefore,

(40) $$\begin{array}{cc} E\frac{V_{\ell_{n}}^{2}}{X_{\ell_{n}}} & \leq \sum_{k=1}^{\ell_{n}}E\left[\frac{1}{X_{k-1}}\sum_{i=1}^{X_{k-1}}{\left(Y_{k,i}-m\right)}^{2}\right]=\sigma^{2}\ell_{n}\leq Cn, \end{array}$$

(41) $$\begin{array}{cc} E\frac{{\left(M_{\ell_{n}}^{H}\right)}^{2}}{X_{\ell_{n}}} & \leq \ell_{n}\sum_{k=1}^{\ell_{n}}E\left[\frac{{\left(X_{k}-mX_{k-1}\right)}^{2}}{X_{k-1}}\right]=\sigma^{2}\ell_{n}^{2}\leq Cn^{2}. \end{array}$$

Since $EV_{r_{n}}^{2}=\sigma^{2}\left(m^{r_{n}}-1\right)/\left(m-1\right)≍m^{r_{n}}$, Markov’s inequality and (40) and (41) yield

(42) $$\begin{array}{cc} \; & P\left(|M_{\ell_{n}}^{H}|>\eta_{n}{\left\{X_{\ell_{n}}EV_{r_{n}}^{2}\right\}}^{1/2}\right)+P\left(V_{\ell_{n}}^{2}>\eta_{n}X_{\ell_{n}}EV_{r_{n}}^{2}\right)\\ \; & \;\leq C\left\{n^{2}e^{2\gamma n}+ne^{\gamma n}\right\}m^{-r_{n}}\leq C\exp\left\{-cn\right\}, \end{array}$$

where the last inequality follows from $r_{n}\geq n/2$ and γ<logm/8.

For all sufficiently large *n*, one has $0<\eta_{n}\leq 1/2$. Applying Lemma 1 conditionally on $F_{\ell_{n}}$, with $N=X_{\ell_{n}}$, $r=r_{n}$, and $\eta =\eta_{n}$, gives

(43) $$\begin{array}{cc} \; & P(\left\{\left|\sum_{j=1}^{X_{\ell_{n}}}{\left(M_{r_{n}}^{H,(j)}\right)}^{2}-X_{\ell_{n}}EV_{r_{n}}^{2}\right|>\eta_{n}X_{\ell_{n}}EV_{r_{n}}^{2}\right\}\\ \; & \;\cup \left\{\left|\sum_{j=1}^{X_{\ell_{n}}}{\left(V_{r_{n}}^{\left(j\right)}\right)}^{2}-X_{\ell_{n}}EV_{r_{n}}^{2}\right|>\eta_{n}X_{\ell_{n}}EV_{r_{n}}^{2}\right\}\;|\;F_{\ell_{n}})\\ \; & \;\leq C\eta_{n}^{-(1+\rho /2)}X_{\ell_{n}}^{-\rho /2}. \end{array}$$

We next apply Lemma 2 conditionally on a fixed population size. By (26), the standardized (2+ρ)th absolute moment of $M_{r_{n}}^{H}$ is bounded uniformly in $r_{n}$. Moreover,

$$\underset{t\geq e^{a_{0}\ell_{n}}}{\sup}\frac{2\varepsilon_{0}\sqrt{n}+2}{\sqrt{t}}\leq \left(2\varepsilon_{0}\sqrt{n}+2\right)e^{-a_{0}\ell_{n}/2}⟶0.$$

Consequently, for all sufficiently large *n*, one has $e^{a_{0}\ell_{n}}\geq N_{0}$ and $2\varepsilon_{0}\sqrt{n}+2\leq c_{1}\sqrt{t}$ for every integer $t\geq e^{a_{0}\ell_{n}}$, where $N_{0}$ and $c_{1}$ are the constants in Lemma 2. Therefore, the lemma applies uniformly over all such *t*, with constants independent of $r_{n}$. Applying it to $M_{r_{n}}^{H}$ and $-M_{r_{n}}^{H}$ yields

(44) $$\begin{array}{cc} \; & \left|\log\frac{P\left(\pm \sum_{j=1}^{t}M_{r_{n}}^{H,(j)}\geq y{\left\{\sum_{j=1}^{t}{\left(M_{r_{n}}^{H,(j)}\right)}^{2}\right\}}^{1/2}\right)}{1-\Phi (y)}\right|\\ \; & \;\leq C\frac{1+y^{2+\rho }}{t^{\rho /2}}\leq C\left(1+y^{2+\rho }\right)e^{-\rho a_{0}\ell_{n}/2}\leq C\left(1+y^{2+\rho }\right)e^{-cn}, \end{array}$$

uniformly for $t\geq e^{a_{0}\ell_{n}}$ and $0\leq y\leq 2\varepsilon_{0}\sqrt{n}+2$. Here, the variables in (44) are *t* independent copies of $M_{r_{n}}^{H}$. Equivalently, (44) yields the uniform exponential bounds

$$\begin{array}{cc} \; & \left(1-\Phi \left(y\right)\right)\exp\left\{-C\left(1+y^{2+\rho }\right)e^{-cn}\right\}\leq P\left(\begin{array}{c} \pm \sum_{j=1}^{t}M_{r_{n}}^{H,(j)}\geq y{\left\{\sum_{j=1}^{t}{\left(M_{r_{n}}^{H,(j)}\right)}^{2}\right\}}^{1/2} \end{array}\right)\\ \; & \;\leq \left(1-\Phi \left(y\right)\right)\exp\left\{C\left(1+y^{2+\rho }\right)e^{-cn}\right\}. \end{array}$$

For every such integer *t*, the branching property and conditional independence give the following, a.s. on $\left\{X_{\ell_{n}}=t\right\}$:

$$\begin{array}{cc} & P\left(\left.\pm \sum_{j=1}^{X_{\ell_{n}}}M_{r_{n}}^{H,(j)}\geq y{\left\{\sum_{j=1}^{X_{\ell_{n}}}{\left(M_{r_{n}}^{H,(j)}\right)}^{2}\right\}}^{1/2}\;\right|\;F_{\ell_{n}}\right)\\ & \;=P\left(\pm \sum_{j=1}^{t}M_{r_{n}}^{H,(j)}\geq y{\left\{\sum_{j=1}^{t}{\left(M_{r_{n}}^{H,(j)}\right)}^{2}\right\}}^{1/2}\right). \end{array}$$

Since $\left\{X_{\ell_{n}}\geq e^{a_{0}\ell_{n}}\right\}\in F_{\ell_{n}}$, the tower property and the preceding uniform bounds give

$$\begin{array}{cc} \; & \left(1-\Phi \left(y\right)\right)\exp\left\{-C\left(1+y^{2+\rho }\right)e^{-cn}\right\}P\left(X_{\ell_{n}}\geq e^{a_{0}\ell_{n}}\right)\\ \; & \;\leq E\left[1_{\left\{X_{\ell_{n}}\geq e^{a_{0}\ell_{n}}\right\}}P\left(\left.\pm \sum_{j=1}^{X_{\ell_{n}}}M_{r_{n}}^{H,(j)}\geq y{\left\{\sum_{j=1}^{X_{\ell_{n}}}{\left(M_{r_{n}}^{H,(j)}\right)}^{2}\right\}}^{1/2}\;\right|\;F_{\ell_{n}}\right)\right]\\ \; & \;=P\left(\pm \sum_{j=1}^{X_{\ell_{n}}}M_{r_{n}}^{H,(j)}\geq y{\left\{\sum_{j=1}^{X_{\ell_{n}}}{\left(M_{r_{n}}^{H,(j)}\right)}^{2}\right\}}^{1/2},X_{\ell_{n}}\geq e^{a_{0}\ell_{n}}\right)\\ \; & \;\leq \left(1-\Phi \left(y\right)\right)\exp\left\{C\left(1+y^{2+\rho }\right)e^{-cn}\right\}P\left(X_{\ell_{n}}\geq e^{a_{0}\ell_{n}}\right). \end{array}$$

For all sufficiently large *n*, (39) gives $P(X_{\ell_{n}}<e^{a_{0}\ell_{n}})\leq 1/2$; hence,

$$\begin{array}{cc} \left|\log P(X_{\ell_{n}}\geq e^{a_{0}\ell_{n}})\right| & =-\log\left(1-P(X_{\ell_{n}}<e^{a_{0}\ell_{n}})\right)\\ \; & \leq 2P\left(X_{\ell_{n}}<e^{a_{0}\ell_{n}}\right)\leq C\exp\left\{-cn\right\}. \end{array}$$

Taking the logarithms in the preceding two-sided bound then gives

(45) $$\begin{array}{cc} \; & \left|\log\frac{P\left(\pm \sum_{j=1}^{X_{\ell_{n}}}M_{r_{n}}^{H,(j)}\geq y{\left\{\sum_{j=1}^{X_{\ell_{n}}}{\left(M_{r_{n}}^{H,(j)}\right)}^{2}\right\}}^{1/2},X_{\ell_{n}}\geq e^{a_{0}\ell_{n}}\right)}{1-\Phi (y)}\right|\\ \; & \;\leq C\left(1+y^{2+\rho }\right)e^{-cn}+\left|\log P(X_{\ell_{n}}\geq e^{a_{0}\ell_{n}})\right|\leq C\left(1+y^{2+\rho }\right)e^{-cn}. \end{array}$$

Put

(46) $$\begin{array}{cc} G_{n}= & \left\{X_{\ell_{n}}\geq e^{a_{0}\ell_{n}}\right\}\\ \; & \cap \left\{|M_{\ell_{n}}^{H}|\leq \eta_{n}{\left\{X_{\ell_{n}}EV_{r_{n}}^{2}\right\}}^{1/2},\;V_{\ell_{n}}^{2}\leq \eta_{n}X_{\ell_{n}}EV_{r_{n}}^{2}\right\}\\ \; & \cap \left\{\left|\sum_{j=1}^{X_{\ell_{n}}}{\left(M_{r_{n}}^{H,(j)}\right)}^{2}-X_{\ell_{n}}EV_{r_{n}}^{2}\right|\leq \eta_{n}X_{\ell_{n}}EV_{r_{n}}^{2},\right.\\ \; & \;\left.\left|\sum_{j=1}^{X_{\ell_{n}}}{\left(V_{r_{n}}^{\left(j\right)}\right)}^{2}-X_{\ell_{n}}EV_{r_{n}}^{2}\right|\leq \eta_{n}X_{\ell_{n}}EV_{r_{n}}^{2}\right\}. \end{array}$$

Equations (39), (42), and (43), together with the tower property, imply

(47) $$\begin{array}{cc} P(G_{n}^{c}) & \leq Ce^{-cn}+C\eta_{n}^{-(1+\rho /2)}e^{-\rho a_{0}\ell_{n}/2}\\ \; & =Ce^{-cn}+C\exp\left\{\left(1+\rho /2\right)\gamma n-\rho a_{0}\ell_{n}/2\right\}\leq Ce^{-\beta_{0}n}, \end{array}$$

where $\ell_{n}\geq n/3$ and $\left(1+\rho /2\right)\gamma =\rho a_{0}/24$, so the second term is at most $Ce^{-\beta_{0}n}$, where $\beta_{0}=\rho a_{0}/8$. The exponents in (39) and (42) satisfy $a_{0}/3>\beta_{0}$ and $\left(\log m\right)/2-2\gamma >\beta_{0}$, respectively. Hence, the polynomial factors in (42) can be absorbed.

Mills’ inequality gives

$$1-\Phi \left(y\right)\geq \frac{c}{1+y}\exp\left\{-\frac{y^{2}}{2}\right\},\;y\geq 0.$$

Our choice gives $0<\varepsilon_{0}<1$ and $\varepsilon_{0}^{2}=\beta_{0}/16<\beta_{0}/8$. Mills’ inequality gives the following, uniformly for $0\leq y\leq 2\varepsilon_{0}\sqrt{n}+2$:

(48) $$\frac{P(G_{n}^{c})}{1-\Phi (y)}\leq C\left(1+y\right)\exp\left\{-\beta_{0}n+\frac{y^{2}}{2}\right\}\leq Ce^{-\beta_{1}n}$$

with $\beta_{1}=\beta_{0}/2$, for all sufficiently large *n*. On $G_{n}$,

(49) $$\begin{array}{cc} \; & \left|\frac{{\left\{\sum_{j=1}^{X_{\ell_{n}}}{\left(M_{r_{n}}^{H,(j)}\right)}^{2}\right\}}^{1/2}}{{\left\{\sum_{j=1}^{X_{\ell_{n}}}{\left(V_{r_{n}}^{\left(j\right)}\right)}^{2}\right\}}^{1/2}}-1\right|\leq C\eta_{n}, \end{array}$$

(50) $$\begin{array}{cc} \; & \frac{|M_{\ell_{n}}^{H}|}{{\left\{\sum_{j=1}^{X_{\ell_{n}}}{\left(V_{r_{n}}^{\left(j\right)}\right)}^{2}\right\}}^{1/2}}+\frac{V_{\ell_{n}}^{2}}{\sum_{j=1}^{X_{\ell_{n}}}{\left(V_{r_{n}}^{\left(j\right)}\right)}^{2}}\leq C\eta_{n}. \end{array}$$

Using (38), we have the following on $G_{n}$:

$$W_{n}^{H}=\frac{\frac{\sum_{j=1}^{X_{\ell_{n}}}M_{r_{n}}^{H,(j)}}{{\left\{\sum_{j=1}^{X_{\ell_{n}}}{\left(V_{r_{n}}^{\left(j\right)}\right)}^{2}\right\}}^{1/2}}+\frac{M_{\ell_{n}}^{H}}{{\left\{\sum_{j=1}^{X_{\ell_{n}}}{\left(V_{r_{n}}^{\left(j\right)}\right)}^{2}\right\}}^{1/2}}}{{\left(1+\frac{V_{\ell_{n}}^{2}}{\sum_{j=1}^{X_{\ell_{n}}}{\left(V_{r_{n}}^{\left(j\right)}\right)}^{2}}\right)}^{1/2}}.$$

On $G_{n}$, (50) gives

$$1\leq {\left(1+\frac{V_{\ell_{n}}^{2}}{\sum_{j=1}^{X_{\ell_{n}}}{\left(V_{r_{n}}^{\left(j\right)}\right)}^{2}}\right)}^{1/2}\leq 1+C\eta_{n}.$$

Together with (49), this gives

$$\begin{array}{cc} \; & \left|\frac{{\left\{\sum_{j=1}^{X_{\ell_{n}}}{\left(M_{r_{n}}^{H,(j)}\right)}^{2}\right\}}^{1/2}}{{\left\{\sum_{j=1}^{X_{\ell_{n}}}{\left(V_{r_{n}}^{\left(j\right)}\right)}^{2}\right\}}^{1/2}{\left(1+\frac{V_{\ell_{n}}^{2}}{\sum_{j=1}^{X_{\ell_{n}}}{\left(V_{r_{n}}^{\left(j\right)}\right)}^{2}}\right)}^{1/2}}-1\right|\leq C\eta_{n},\\ \; & \frac{|M_{\ell_{n}}^{H}|}{{\left\{\sum_{j=1}^{X_{\ell_{n}}}{\left(V_{r_{n}}^{\left(j\right)}\right)}^{2}\right\}}^{1/2}{\left(1+\frac{V_{\ell_{n}}^{2}}{\sum_{j=1}^{X_{\ell_{n}}}{\left(V_{r_{n}}^{\left(j\right)}\right)}^{2}}\right)}^{1/2}}\leq C\eta_{n}. \end{array}$$

Consequently, for some $C_{*}>0$, we have the following uniformly on $G_{n}$:

$$\left|W_{n}^{H}-\frac{\sum_{j=1}^{X_{\ell_{n}}}M_{r_{n}}^{H,(j)}}{{\left\{\sum_{j=1}^{X_{\ell_{n}}}{\left(M_{r_{n}}^{H,(j)}\right)}^{2}\right\}}^{1/2}}\right|\leq C_{*}\eta_{n}\left(1+\left|\frac{\sum_{j=1}^{X_{\ell_{n}}}M_{r_{n}}^{H,(j)}}{{\left\{\sum_{j=1}^{X_{\ell_{n}}}{\left(M_{r_{n}}^{H,(j)}\right)}^{2}\right\}}^{1/2}}\right|\right).$$

Choose $K>4C_{*}$ and put

$$\delta_{n,x}=K\left(1+x\right)\eta_{n},\;0\leq x\leq \varepsilon_{0}\sqrt{n}.$$

For all sufficiently large *n*, $C_{*}\eta_{n}\leq 1/2$, and we have the following uniformly for $0\leq x\leq \varepsilon_{0}\sqrt{n}$:

$$\delta_{n,x}-C_{*}\eta_{n}\left(1+x+\delta_{n,x}\right)=\left(1+x\right)\eta_{n}\left\{K-C_{*}-C_{*}K\eta_{n}\right\}>0.$$

Since $C_{*}\eta_{n}<1$, both comparison bounds are increasing in the displayed self-normalized ratio. If this ratio is at least $x+\delta_{n,x}$ on $G_{n}$, then the preceding comparison gives

$$W_{n}^{H}\geq x+\delta_{n,x}-C_{*}\eta_{n}\left(1+x+\delta_{n,x}\right)>x.$$

Conversely, suppose that $W_{n}^{H}\geq x$, while the displayed ratio is smaller than $x-\delta_{n,x}$ on $G_{n}$. Then,

$$W_{n}^{H}<x-\delta_{n,x}+C_{*}\eta_{n}\{1+|x-\delta_{n,x}\left|\right\}\leq x-\delta_{n,x}+C_{*}\eta_{n}\left(1+x+\delta_{n,x}\right)<x,$$

which is a contradiction. Thus, we have

(51) $$\begin{array}{c} \left\{\sum_{j=1}^{X_{\ell_{n}}}M_{r_{n}}^{H,(j)}\geq \left(x+\delta_{n,x}\right){\left\{\sum_{j=1}^{X_{\ell_{n}}}{\left(M_{r_{n}}^{H,(j)}\right)}^{2}\right\}}^{1/2}\right\}\cap G_{n}\;\;\;\;\;\;\;\;\;\;\;\;\;\;\;\;\;\;\;\;\;\;\;\;\\ \subseteq \left\{W_{n}^{H}\geq x\right\}\cap G_{n}\subseteq \\ \;\;\;\;\;\;\;\;\;\;\;\;\;\;\;\;\;\;\;\;\;\;\;\left\{\sum_{j=1}^{X_{\ell_{n}}}M_{r_{n}}^{H,(j)}\geq \left(x-\delta_{n,x}\right){\left\{\sum_{j=1}^{X_{\ell_{n}}}{\left(M_{r_{n}}^{H,(j)}\right)}^{2}\right\}}^{1/2}\right\}\cap G_{n}. \end{array}$$

For all sufficiently large *n*, uniformly over $4K\eta_{n}\leq x\leq \varepsilon_{0}\sqrt{n}$, it holds that

$$0\leq x-\delta_{n,x}\leq x+\delta_{n,x}\leq 2\varepsilon_{0}\sqrt{n}+2,\;1+{\left(x\pm \delta_{n,x}\right)}^{2+\rho }\leq C\left(1+x^{2+\rho }\right).$$

Therefore, taking the probabilities in (51) gives

(52) $$\begin{array}{cc} \; & P\left(\sum_{j=1}^{X_{\ell_{n}}}M_{r_{n}}^{H,(j)}\geq \left(x+\delta_{n,x}\right){\left\{\sum_{j=1}^{X_{\ell_{n}}}{\left(M_{r_{n}}^{H,(j)}\right)}^{2}\right\}}^{1/2},X_{\ell_{n}}\geq e^{a_{0}\ell_{n}}\right)-P\left(G_{n}^{c}\right)\\ \; & \;\leq P(W_{n}^{H}\geq x)\\ \; & \;\leq P\left(\sum_{j=1}^{X_{\ell_{n}}}M_{r_{n}}^{H,(j)}\geq \left(x-\delta_{n,x}\right){\left\{\sum_{j=1}^{X_{\ell_{n}}}{\left(M_{r_{n}}^{H,(j)}\right)}^{2}\right\}}^{1/2},X_{\ell_{n}}\geq e^{a_{0}\ell_{n}}\right)+P\left(G_{n}^{c}\right). \end{array}$$

Mills’ inequality further yields

(53) $$\begin{array}{cc} \left|\log\frac{1-\Phi (x\pm \delta_{n,x})}{1-\Phi (x)}\right| & \leq C\left(1+x\right)\delta_{n,x}\leq C{\left(1+x\right)}^{2}e^{-\gamma n}\leq C\left(1+x^{2+\rho }\right)e^{-\gamma n}. \end{array}$$

Combining (45), (48), (52), and (53) together and using the inequality |log(1+v)|≤2|v| for |v|≤1/2, we obtain

(54) $$\left|\log\frac{P(W_{n}^{H}\geq x)}{1-\Phi (x)}\right|\leq C\left(1+x^{2+\rho }\right)e^{-cn},\;4K\eta_{n}\leq x\leq \varepsilon_{0}\sqrt{n}.$$

It remains to consider the case $0\leq x<4K\eta_{n}$. For all sufficiently large *n*, $\delta_{n,x}\leq 2K\eta_{n}$; hence, $x+\delta_{n,x}<6K\eta_{n}$. Thus, after intersection with $G_{n}$, the event in which the future self-normalized ratio is at least $6K\eta_{n}$ is contained in $\left\{W_{n}^{H}\geq x\right\}$. On the other hand, if the future self-normalized ratio is at most $-6K\eta_{n}$, then the preceding comparison gives

$$W_{n}^{H}\leq -6K\eta_{n}+C_{*}\eta_{n}\left(1+6K\eta_{n}\right)<0\leq x$$

on $G_{n}$. Therefore, it holds that

(55) $$\begin{array}{cc} P(W_{n}^{H}\geq x) & \geq P\left(\begin{array}{c} \sum_{j=1}^{X_{\ell_{n}}}M_{r_{n}}^{H,(j)}\geq 6K\eta_{n}{\left\{\sum_{j=1}^{X_{\ell_{n}}}{\left(M_{r_{n}}^{H,(j)}\right)}^{2}\right\}}^{1/2},\;X_{\ell_{n}}\geq e^{a_{0}\ell_{n}} \end{array}\right)-P\left(G_{n}^{c}\right),\\ P(W_{n}^{H}\geq x) & \leq P\left(X_{\ell_{n}}\geq e^{a_{0}\ell_{n}}\right)+P\left(G_{n}^{c}\right)\\ \; & -P\left(\begin{array}{c} -\sum_{j=1}^{X_{\ell_{n}}}M_{r_{n}}^{H,(j)}\geq 6K\eta_{n}{\left\{\sum_{j=1}^{X_{\ell_{n}}}{\left(M_{r_{n}}^{H,(j)}\right)}^{2}\right\}}^{1/2},X_{\ell_{n}}\geq e^{a_{0}\ell_{n}} \end{array}\right). \end{array}$$

By (45), for either sign we have

$$\begin{array}{cc} \; & P\left(\begin{array}{c} \pm \sum_{j=1}^{X_{\ell_{n}}}M_{r_{n}}^{H,(j)}\geq 6K\eta_{n}{\left\{\sum_{j=1}^{X_{\ell_{n}}}{\left(M_{r_{n}}^{H,(j)}\right)}^{2}\right\}}^{1/2},X_{\ell_{n}}\geq e^{a_{0}\ell_{n}} \end{array}\right)\\ \; & \;\;\;\;=\left\{1-\Phi (6K\eta_{n})\right\}\exp\left\{O(e^{-cn})\right\}=\frac{1}{2}+O\left(e^{-cn}\right), \end{array}$$

where the last equality uses $\eta_{n}=e^{-\gamma n}$. Together with (39), (48), and $1-\Phi \left(x\right)=1/2+O\left(e^{-\gamma n}\right)$ on this interval, (55) implies

(56) $$\left|\log\frac{P(W_{n}^{H}\geq x)}{1-\Phi (x)}\right|\leq Ce^{-cn},\;0\leq x<4K\eta_{n}.$$

Combining (54) and (56) gives

(57) $$\underset{0\leq x\leq \varepsilon_{0}\sqrt{n}}{\sup}\frac{1}{1+x^{2+\rho }}\left|\log\frac{P(W_{n}^{H}\geq x)}{1-\Phi (x)}\right|\leq Ce^{-cn}.$$

By (7),

$$1-\frac{{\left(W_{n}^{H}\right)}^{2}}{S_{n-1}}=\frac{{\hat{V}}_{n}^{2}}{V_{n}^{2}},\;T_{n}^{H}=\frac{W_{n}^{H}}{\sqrt{1-{\left(W_{n}^{H}\right)}^{2}/S_{n-1}}}.$$

For fixed $S_{n-1}$, the right-hand side, viewed as a function of $W_{n}^{H}$, is odd and strictly increasing on $\left(-\sqrt{S_{n-1}},\sqrt{S_{n-1}}\right)$. Hence, for x≥0,

(58) $$\left\{T_{n}^{H}\geq x\right\}=\left\{W_{n}^{H}\geq \frac{x}{\sqrt{1+x^{2}/S_{n-1}}}\right\}.$$

On $\left\{X_{\ell_{n}}\geq e^{a_{0}\ell_{n}}\right\}$, one has $S_{n-1}\geq X_{\ell_{n}}\geq e^{a_{0}\ell_{n}}$. Hence, the random threshold in (58) lies between $x/\sqrt{1+x^{2}e^{-a_{0}\ell_{n}}}$ and *x*; therefore,

(59) $$P\left(W_{n}^{H}\geq x\right)\leq P\left(T_{n}^{H}\geq x\right)\leq P\left(W_{n}^{H}\geq \frac{x}{\sqrt{1+x^{2}e^{-a_{0}\ell_{n}}}}\right)+P\left(X_{\ell_{n}}<e^{a_{0}\ell_{n}}\right).$$

We have

(60) $$\begin{array}{cc} 0\leq x-\frac{x}{\sqrt{1+x^{2}e^{-a_{0}\ell_{n}}}} & \leq Cx^{3}e^{-a_{0}\ell_{n}},\\ \left|\log\frac{1-\Phi \left(x/\sqrt{1+x^{2}e^{-a_{0}\ell_{n}}}\right)}{1-\Phi (x)}\right| & \leq C\left(1+x\right)\left(x-\frac{x}{\sqrt{1+x^{2}e^{-a_{0}\ell_{n}}}}\right)\\ \; & \leq C\left(x^{3}+x^{4}\right)e^{-a_{0}\ell_{n}}\leq C\left(1+x^{2+\rho }\right)e^{-b_{0}n} \end{array}$$

uniformly for $0\leq x\leq \varepsilon_{0}\sqrt{n}$, where $b_{0}>0$. Indeed, since 0<ρ≤1 and $\ell_{n}\geq n/3$,

$$x^{3}+x^{4}\leq C\left(1+x^{2+\rho }\right)n^{1-\rho /2},\;n^{1-\rho /2}e^{-a_{0}\ell_{n}}\leq Ce^{-b_{0}n}.$$

The same normal tail bound, (39), and the choice of $\varepsilon_{0}$ also give

(61) $$\underset{0\leq x\leq \varepsilon_{0}\sqrt{n}}{\sup}\frac{P(X_{\ell_{n}}<e^{a_{0}\ell_{n}})}{1-\Phi (x)}\leq Ce^{-cn}.$$

Combining (57), (59), (60), and (61) together and using the inequality |log(1+v)|≤2|v| for |v|≤1/2 yields constants $C_{+},b_{+}>0$ such that

$$\left|\log\frac{P(T_{n}^{H}\geq x)}{1-\Phi (x)}\right|\leq C_{+}\left(1+x^{2+\rho }\right)e^{-b_{+}n},\;0\leq x\leq \varepsilon_{0}\sqrt{n}.$$

The same argument holds with $M_{\ell_{n}}^{H}$ and ${\left(M_{r_{n}}^{H,(j)}\right)}_{j\geq 1}$ replaced by their negatives. Thus, for some constants $C_{-},b_{-}>0$,

$$\left|\log\frac{P(T_{n}^{H}\leq -x)}{\Phi (-x)}\right|\leq C_{-}\left(1+x^{2+\rho }\right)e^{-b_{-}n},\;0\leq x\leq \varepsilon_{0}\sqrt{n}.$$

Here, one may take $b_{+}=b_{-}=\gamma$.

Taking

$$c:=\frac{1}{2}\min\left\{\varepsilon_{0},b_{+},b_{-}\right\},$$

we have the following for both signs:

(62) $$\underset{0\leq x\leq c\sqrt{n}}{\sup}\left(1+x^{2+\rho }\right)e^{-b_{\pm }n}\leq Cn^{1+\rho /2}e^{-b_{\pm }n}\leq Ce^{-cn}.$$

Consequently, it holds that

$$\underset{0\leq x\leq c\sqrt{n}}{\sup}\left|\log\frac{P(T_{n}^{H}\geq x)}{1-\Phi (x)}\right|+\underset{0\leq x\leq c\sqrt{n}}{\sup}\left|\log\frac{P(T_{n}^{H}\leq -x)}{\Phi (-x)}\right|\leq Ce^{-cn}.$$

This completes the proof of theorem. □

### 5.3. Proof of Theorem 2

**Proof.**  We first give a proof for (14). Put ℓ=⌊n/2⌋, r=n−ℓ, $N=X_{\ell }$, and

$$\begin{array}{c} \tau_{r}^{2}=\sigma^{2}\frac{m^{r}-1}{m-1},\\ H_{n}=\left\{N\geq e^{\alpha \ell }\right\},\;\eta_{n}=e^{-\gamma_{S}n}. \end{array}$$

All conditional independence and moment estimates below are under P. Conditional on $F_{\ell }$, the *N* descendant trees are independent copies of the original process. Hence,

(63) $$\begin{array}{cc} M_{n}^{H} & =M_{\ell }^{H}+\sum_{j=1}^{N}M_{r}^{H,(j)},\\ V_{n}^{2} & =V_{\ell }^{2}+\sum_{j=1}^{N}{\left(V_{r}^{\left(j\right)}\right)}^{2}. \end{array}$$

On {N>0}, write

(64) $$Z_{n}=\frac{\sum_{j=1}^{N}M_{r}^{H,(j)}}{\tau_{r}\sqrt{N}}.$$

By Lemmas 1 and 5, we get

$$\underset{z\in R}{\sup}\left|P\left(Z_{n}\leq z|F_{\ell }\right)-\Phi \left(z\right)\right|\leq CN^{-\rho /2}.$$

The constants in this estimate are independent of *r*. Using (33), on $H_{n}$ we obtain

$$\underset{z\in R}{\sup}\left|P_{S}\left(Z_{n}\leq z|F_{\ell }\right)-\Phi \left(z\right)\right|\leq Ce^{-\rho \alpha \ell /2}+q^{e^{\alpha \ell }}.$$

For all sufficiently large *n*, integration under $P_{S}$ and (32) gives

(65) $$\begin{array}{cc} \underset{z\in R}{\sup}\left|P_{S}\left(Z_{n}\leq z\right)-\Phi \left(z\right)\right| & \leq Ce^{-\rho \alpha \ell /2}+q^{e^{\alpha \ell }}+e^{-\alpha \ell }\\ \; & \leq Ce^{-4\gamma_{S}n}, \end{array}$$

where we have used $\rho \alpha /4=\left(4+\rho \right)\gamma_{S}$.

We next estimate the contribution of the first *ℓ* generations. Since ℓ≤r, $\tau_{\ell }^{2}\leq \tau_{r}^{2}$. By (25) and $P_{S}\left(A\right)\leq P\left(A\right)/\left(1-q\right)$,

(66) $$\begin{array}{cc} P_{S}\left(H_{n},\;\left|M_{\ell }^{H}\right|>\eta_{n}\tau_{r}\sqrt{N}\right) & \leq C\eta_{n}^{-2}e^{-\alpha \ell }, \end{array}$$

(67) $$\begin{array}{cc} P_{S}\left(H_{n},\;V_{\ell }^{2}>\eta_{n}N\tau_{r}^{2}\right) & \leq C\eta_{n}^{-1}e^{-\alpha \ell }. \end{array}$$

Conditioning under P and using (27), we similarly obtain

(68) $$P_{S}\left(H_{n},\;\left|\frac{\sum_{j=1}^{N}{\left(V_{r}^{\left(j\right)}\right)}^{2}}{N\tau_{r}^{2}}-1\right|>\eta_{n}\right)\leq C\eta_{n}^{-(1+\rho /2)}e^{-\rho \alpha \ell /2}.$$

Moreover, $Z_{n}$ has conditional mean zero and conditional variance one under P; therefore, Chebyshev’s inequality yields

(69) $$P_{S}\left(H_{n},\;\left|Z_{n}\right|>\frac{1}{2}\sqrt{\eta_{n}N}\right)\leq C\eta_{n}^{-1}e^{-\alpha \ell }.$$

Let $G_{n}$ be the intersection of $H_{n}$ with the four events

$$\begin{array}{c} |M_{\ell }^{H}|\leq \eta_{n}\tau_{r}\sqrt{N},\;V_{\ell }^{2}\leq \eta_{n}N\tau_{r}^{2},\\ \left|\frac{\sum_{j=1}^{N}{\left(V_{r}^{\left(j\right)}\right)}^{2}}{N\tau_{r}^{2}}-1\right|\leq \eta_{n},\;\left|Z_{n}\right|\leq \frac{1}{2}\sqrt{\eta_{n}N}. \end{array}$$

Equations (32) and (66)–(69) imply

(70) $$\begin{array}{cc} P_{S}\left(G_{n}^{c}\right) & \leq Ce^{-\alpha \ell }+C\eta_{n}^{-2}e^{-\alpha \ell }+C\eta_{n}^{-(1+\rho /2)}e^{-\rho \alpha \ell /2}\\ \; & \leq Ce^{-3\gamma_{S}n}. \end{array}$$

Indeed, ℓ≥n/2−1 and

$$\frac{\alpha }{2}-2\gamma_{S}=\frac{8}{\rho }\gamma_{S},\;\frac{\rho \alpha }{4}-\left(1+\frac{\rho }{2}\right)\gamma_{S}=\left(3+\frac{\rho }{2}\right)\gamma_{S}.$$

On $G_{n}$, the branching decomposition gives

$$\left|\frac{M_{n}^{H}}{\tau_{r}\sqrt{N}}-Z_{n}\right|\leq \eta_{n},\;\left|\frac{M_{n}^{H}}{\tau_{r}\sqrt{N}}\right|\leq \frac{1}{2}\sqrt{\eta_{n}N}+\eta_{n}\leq \sqrt{\eta_{n}N}$$

for all sufficiently large *n*, since $\eta_{n}\leq 1/4$. As $S_{n-1}\geq N$, the variance identity yields

(71) $$\begin{array}{c} \frac{{\hat{V}}_{n}^{2}}{N\tau_{r}^{2}}=\frac{V_{n}^{2}}{N\tau_{r}^{2}}-\frac{{\left(M_{n}^{H}\right)}^{2}}{N\tau_{r}^{2}S_{n-1}},\\ 1-2\eta_{n}\leq \frac{{\hat{V}}_{n}^{2}}{N\tau_{r}^{2}}\leq 1+2\eta_{n}. \end{array}$$

Since $T_{n}^{H}=M_{n}^{H}/{\hat{V}}_{n}$, these bounds, (65), and (70) give the following for every z∈R:

(72) $$\begin{array}{cc} P_{S}\left(T_{n}^{H}\leq z\right) & \geq \Phi \;\left(\min\left\{z\sqrt{1-2\eta_{n}},z\sqrt{1+2\eta_{n}}\right\}-\eta_{n}\right)-Ce^{-3\gamma_{S}n},\\ P_{S}\left(T_{n}^{H}\leq z\right) & \leq \Phi \;\left(\max\left\{z\sqrt{1-2\eta_{n}},z\sqrt{1+2\eta_{n}}\right\}+\eta_{n}\right)+Ce^{-3\gamma_{S}n}. \end{array}$$

By Lemma 5, the two expressions involving Φ differ uniformly from Φ(z) by at most $C\eta_{n}$. Consequently, it holds that

$$\underset{z\in R}{\sup}\left|P_{S}\left(T_{n}^{H}\leq z\right)-\Phi \left(z\right)\right|\leq C\eta_{n}+Ce^{-3\gamma_{S}n}\leq Ce^{-\gamma_{S}n}.$$

This proves (14) under $P_{S}$.

To pass to conditioning on $X_{n}>0$, put $q_{n}=f^{\circ n}\left(0\right)$. Since $f^{\prime }$ is increasing on [0,q],

$$0\leq q-q_{n+1}=f\left(q\right)-f\left(q_{n}\right)\leq d\left(q-q_{n}\right),\;q-q_{n}\leq qd^{n}.$$

For every event *A*, the inclusion $S\subset \{X_{n}>0\}$ and the conditional mixture identity yield

(73) $$\left|P_{n}^{+}\left(A\right)-P_{S}\left(A\right)\right|\leq \frac{q-q_{n}}{1-q_{n}}\leq \frac{q}{1-q}d^{n}.$$

As $d<\left(1+d\right)/2=e^{-2\alpha }$ and $2\alpha >\gamma_{S}$, (14) also holds under $P_{n}^{+}$.

It remains to prove (13). Let $Q_{n}$ denote either $P_{S}$ or $P_{n}^{+}$. By continuity of Φ, the Kolmogorov bound also controls strict inequalities. Hence, for either sign,

$$\left|Q_{n}\left(\pm T_{n}^{H}\geq x\right)-\left(1-\Phi \left(x\right)\right)\right|\leq Ce^{-\gamma_{S}n},\;x\geq 0.$$

Using the normal tail bound

$$1-\Phi \left(x\right)\geq \frac{c}{1+x}e^{-x^{2}/2},\;x\geq 0,$$

we obtain the following uniformly for $0\leq x\leq \sqrt{\gamma_{S}n}$:

(74) $$\begin{array}{cc} \left|\frac{Q_{n}\left(\pm T_{n}^{H}\geq x\right)}{1-\Phi (x)}-1\right| & \leq C\left(1+x\right)e^{-\gamma_{S}n+x^{2}/2}\\ \; & \leq C\left(1+\sqrt{n}\right)e^{-\gamma_{S}n/2}\leq Ce^{-\gamma_{S}n/4}. \end{array}$$

The last bound is at most 1/2 for all sufficiently large *n*. The inequality |log(1+u)|≤2|u| for |u|≤1/2 now gives (13) with $c=\min\{\sqrt{\gamma_{S}},\gamma_{S}/4\}$. The same argument applies to both tails and both conditional measures. □

### 5.4. Proof of Theorem 3

**Proof.**  Put $\ell_{n}=\left\lfloor n/2\right\rfloor$, $r_{n}=n-\ell_{n}$, and $a_{0}=\left(\log k_{0}\right)/2$. Since every individual has at least $k_{0}$ offspring,

$$X_{\ell_{n}}\geq k_{0}^{\ell_{n}}\geq e^{a_{0}\ell_{n}}\;a.s.,\;\log m\geq 2a_{0}.$$

Lemmas 1 and 2 apply under the present assumptions. In the proof of Theorem 1, take

$$\gamma =\frac{\rho a_{0}}{12(2+\rho )},\;\eta_{n}=e^{-\gamma n},\;\varepsilon_{0}=\min\left\{1,\sqrt{\frac{\rho a_{0}}{128}}\right\}.$$

Since $p_{0}=0$, the branching decomposition and the estimates for the early generations remain valid. The event $\left\{X_{\ell_{n}}<e^{a_{0}\ell_{n}}\right\}$ in (39) is empty, and the conditional estimates (43) and (44) hold for every possible value of $X_{\ell_{n}}$. Consequently, with $G_{n}$ defined by (46),

$$P\left(G_{n}^{c}\right)\leq Ce^{-\beta_{0}n},\;\beta_{0}=\frac{\rho a_{0}}{8}.$$

Our choice gives $\varepsilon_{0}^{2}<\beta_{0}/8$. Thus, the comparison with the normal tail and the Studentization argument in the proof of Theorem 1 apply without change; the population remainder in (59) is zero. Thus, for some constants $C_{+},b_{+}>0$,

$$\left|\log\frac{P(T_{n}^{H}\geq x)}{1-\Phi (x)}\right|\leq C_{+}\left(1+x^{2+\rho }\right)e^{-b_{+}n},\;0\leq x\leq \varepsilon_{0}\sqrt{n}.$$

The same argument holds with $M_{\ell_{n}}^{H}$ and ${\left(M_{r_{n}}^{H,(j)}\right)}_{j\geq 1}$ replaced by their negatives. Thus, for some constants $C_{-},b_{-}>0$,

$$\left|\log\frac{P(T_{n}^{H}\leq -x)}{\Phi (-x)}\right|\leq C_{-}\left(1+x^{2+\rho }\right)e^{-b_{-}n},\;0\leq x\leq \varepsilon_{0}\sqrt{n}.$$

Here, one may take $b_{+}=b_{-}=\gamma$. Taking

$$c:=\frac{1}{2}\min\left\{\varepsilon_{0},b_{+},b_{-}\right\},$$

we have the following for both signs:

(75) $$\underset{0\leq x\leq c\sqrt{n}}{\sup}\left(1+x^{2+\rho }\right)e^{-b_{\pm }n}\leq Cn^{1+\rho /2}e^{-b_{\pm }n}\leq Ce^{-cn}.$$

Consequently,

$$\underset{0\leq x\leq c\sqrt{n}}{\sup}\left|\log\frac{P(T_{n}^{H}\geq x)}{1-\Phi (x)}\right|+\underset{0\leq x\leq c\sqrt{n}}{\sup}\left|\log\frac{P(T_{n}^{H}\leq -x)}{\Phi (-x)}\right|\leq Ce^{-cn}.$$

Having proved the stronger two-sided uniform estimate, the assertion follows. □

### 5.5. Proof of Corollary 1

**Proof.**  For fixed y>0, $a_{n}y=o\left(\sqrt{n}\right)$, and Theorem 1 yields

$$\begin{array}{cc} \frac{1}{a_{n}^{2}}\log P\left(T_{n}^{H}\geq a_{n}y\right) & =\frac{1}{a_{n}^{2}}\log\left\{1-\Phi \left(a_{n}y\right)\right\}+O\left(a_{n}^{-2}e^{-cn}\right),\\ \frac{1}{a_{n}^{2}}\log P\left(T_{n}^{H}\leq -a_{n}y\right) & =\frac{1}{a_{n}^{2}}\log\Phi \left(-a_{n}y\right)+O\left(a_{n}^{-2}e^{-cn}\right). \end{array}$$

Since $a_{n}\to \infty$, the error term tends to zero. The standard estimate for the normal tail is

$$\log\left\{1-\Phi \left(t\right)\right\}=-\frac{t^{2}}{2}+O\left(\log t\right),\;t⟶\infty ,$$

which together with Φ(−t)=1−Φ(t) gives

(76) $$\begin{array}{cc} \lim_{n\to \infty }\frac{1}{a_{n}^{2}}\log P\left(T_{n}^{H}\geq a_{n}y\right) & =-\frac{y^{2}}{2}, \end{array}$$

(77) $$\begin{array}{cc} \lim_{n\to \infty }\frac{1}{a_{n}^{2}}\log P\left(T_{n}^{H}\leq -a_{n}y\right) & =-\frac{y^{2}}{2}. \end{array}$$

The upper bound is immediate if B=⌀; otherwise, put

$$r_{B}=\underset{x\in \bar{B}}{\inf}\left|x\right|.$$

If $r_{B}=0$, then the bound follows from $P(T_{n}^{H}/a_{n}\in B)\leq 1$. When $r_{B}>0$, it holds that

$$\begin{array}{cc} P(T_{n}^{H}/a_{n}\in B) & \leq P(|T_{n}^{H}|\geq a_{n}r_{B})\\ & \leq P\left(T_{n}^{H}\geq a_{n}r_{B}\right)+P\left(T_{n}^{H}\leq -a_{n}r_{B}\right). \end{array}$$

Therefore, Equations (76) and (77) give

(78) $$\underset{n\to \infty }{\lim\;\sup}\frac{1}{a_{n}^{2}}\log P\left(T_{n}^{H}/a_{n}\in B\right)\leq -\frac{r_{B}^{2}}{2}=-\underset{x\in \bar{B}}{\inf}\frac{x^{2}}{2}.$$

If $B^{\circ }=⌀$, the lower bound is trivial; otherwise, given η>0, choose $x_{0}\in B^{\circ }$ such that

(79) $$\frac{x_{0}^{2}}{2}\leq \underset{x\in B^{\circ }}{\inf}\frac{x^{2}}{2}+\eta .$$

Assume first that $x_{0}>0$. Choose $0<\delta <x_{0}$ sufficiently small that $\left[x_{0}-\delta ,x_{0}+\delta \right]\subset B^{\circ }$. Then, we have

$$\begin{array}{cc} P(T_{n}^{H}/a_{n}\in B) & \geq P\left(T_{n}^{H}\geq a_{n}\left(x_{0}-\delta \right)\right)-P\left(T_{n}^{H}\geq a_{n}\left(x_{0}+\delta \right)\right). \end{array}$$

By (76), the ratio of the second tail to the first satisfies

$$\frac{1}{a_{n}^{2}}\log\frac{P(T_{n}^{H}\geq a_{n}\left(x_{0}+\delta \right))}{P(T_{n}^{H}\geq a_{n}\left(x_{0}-\delta \right))}⟶-2x_{0}\delta <0.$$

Therefore, the ratio tends to zero, meaning that for all sufficiently large *n*,

$$\begin{array}{cc} P(T_{n}^{H}/a_{n}\in B) & \geq \frac{3}{4}P\left(T_{n}^{H}\geq a_{n}\left(x_{0}-\delta \right)\right). \end{array}$$

Theorem 1 then gives

$$\begin{array}{cc} P(T_{n}^{H}/a_{n}\in B) & \geq \frac{3}{4}\left\{1-\Phi \left(a_{n}\left(x_{0}-\delta \right)\right)\right\}\exp\left\{-Ce^{-cn}\right\}. \end{array}$$

Using the lower Mills bound

$$1-\Phi \left(t\right)\geq \frac{1}{\sqrt{2\pi }\left(1+t\right)}e^{-t^{2}/2},\;t\geq 0,$$

then taking the logarithms and dividing by $a_{n}^{2}$, we obtain

$$\underset{n\to \infty }{\lim\;\inf}\frac{1}{a_{n}^{2}}\log P\left(T_{n}^{H}/a_{n}\in B\right)\geq -\frac{{\left(x_{0}-\delta \right)}^{2}}{2}.$$

If $x_{0}<0$, choose $0<\delta <|x_{0}|$ such that $\left[x_{0}-\delta ,x_{0}+\delta \right]\subset B^{\circ }$. In this case,

$$\begin{array}{cc} P(T_{n}^{H}/a_{n}\in B) & \geq P(T_{n}^{H}\leq -a_{n}\left(\right|x_{0}|-\delta ))-P(T_{n}^{H}\leq -a_{n}\left(\right|x_{0}|+\delta )). \end{array}$$

Equation (77) gives

$$\frac{1}{a_{n}^{2}}\log\frac{P(T_{n}^{H}\leq -a_{n}\left(\right|x_{0}\left|+\delta )\right)}{P(T_{n}^{H}\leq -a_{n}\left(\right|x_{0}\left|-\delta )\right)}⟶-2\left|x_{0}\right|\delta <0.$$

The same argument, using the left tail estimate in Theorem 1 and Φ(−t)=1−Φ(t), gives

$$\underset{n\to \infty }{\lim\;\inf}\frac{1}{a_{n}^{2}}\log P\left(T_{n}^{H}/a_{n}\in B\right)\geq -\frac{\left(\right|x_{0}{\left|-\delta \right)}^{2}}{2}.$$

If $x_{0}=0$, choose δ>0 such that $\left[-\delta ,\delta \right]\subset B^{\circ }$. Then,

$$\begin{array}{cc} P(T_{n}^{H}/a_{n}\in B) & \geq 1-P\left(T_{n}^{H}\geq a_{n}\delta \right)-P\left(T_{n}^{H}\leq -a_{n}\delta \right)⟶1 \end{array}$$

by (76) and (77). Hence, the corresponding logarithmic liminf is non-negative.

Letting δ↓0 in the cases $x_{0}\neq 0$, using (79), and then letting η↓0 gives

(80) $$\underset{n\to \infty }{\lim\;\inf}\frac{1}{a_{n}^{2}}\log P\left(T_{n}^{H}/a_{n}\in B\right)\geq -\underset{x\in B^{\circ }}{\inf}\frac{x^{2}}{2}.$$

Combining (78) and (80), we obtain (9). □

### 5.6. Proof of Corollary 3

**Proof.**  Let $Q_{n}$ denote either $P_{S}$ or $P_{n}^{+}$. For fixed y>0, Theorem 2 and $a_{n}y=o\left(\sqrt{n}\right)$ give the following for both signs:

$$\frac{1}{a_{n}^{2}}\log Q_{n}\left(\pm T_{n}^{H}\geq a_{n}y\right)=\frac{1}{a_{n}^{2}}\log\left\{1-\Phi \left(a_{n}y\right)\right\}+O\left(a_{n}^{-2}e^{-cn}\right)⟶-\frac{y^{2}}{2}.$$

By the Kolmogorov bound in (14) and continuity of Φ, the same limit holds for strict tails. For a Borel set *B*, the upper bound follows by bounding its probability by the two tails at distance $\inf_{x\in \bar{B}}\left|x\right|$ from zero. The lower bound follows by taking an interval inside $B^{\circ }$ and subtracting its endpoint tails, as in the proof of Corollary 1. An interval containing zero has probability tending to one. These arguments apply to the sequence $P_{n}^{+}$ as well as to the fixed measure $P_{S}$, and prove (15). □

### 5.7. Proof of Corollary 2

**Proof.**  Let $c_{*}>0$ be the constant in Theorem 1 and choose

$$0<c<\min\{c_{*},c_{*}^{2}/8\}.$$

Put $h_{n}=c_{*}\sqrt{n}/2$. By Theorem 1, for some C>0, the two logarithmic bounds hold with $Ce^{-cn}$ on $0\leq x\leq c_{*}\sqrt{n}$. Moreover,

(81) $$1-\Phi \left(h_{n}\right)=\Phi \left(-h_{n}\right)\leq C\exp\left\{-\frac{h_{n}^{2}}{2}\right\}=Ce^{-c_{*}^{2}n/8}\leq Ce^{-cn}.$$

Now,

$$\begin{array}{cc} \underset{x\in R}{\sup}\left|P\left(T_{n}^{H}\leq x\right)-\Phi \left(x\right)\right| & \leq \underset{x\geq h_{n}}{\sup}\left|P\left(T_{n}^{H}\leq x\right)-\Phi \left(x\right)\right|+\underset{0\leq x<h_{n}}{\sup}\left|P\left(T_{n}^{H}\leq x\right)-\Phi \left(x\right)\right|\\ \; & \;+\underset{-h_{n}<x\leq 0}{\sup}\left|P\left(T_{n}^{H}\leq x\right)-\Phi \left(x\right)\right|\\ \; & \;+\underset{x\leq -h_{n}}{\sup}\left|P\left(T_{n}^{H}\leq x\right)-\Phi \left(x\right)\right|\\ \; & =:H_{1}+H_{2}+H_{3}+H_{4}. \end{array}$$

By Theorem 1 and (81), we get

(82) $$\begin{array}{cc} H_{1} & \leq P\left(T_{n}^{H}\geq h_{n}\right)+1-\Phi \left(h_{n}\right)\\ \; & \leq \left\{1-\Phi \left(h_{n}\right)\right\}\left[\exp\left\{Ce^{-cn}\right\}+1\right]\\ \; & \leq Ce^{-cn} \end{array}$$

and

(83) $$\begin{array}{cc} H_{4} & \leq P\left(T_{n}^{H}\leq -h_{n}\right)+\Phi \left(-h_{n}\right)\\ \; & \leq \Phi \left(-h_{n}\right)\left[\exp\left\{Ce^{-cn}\right\}+1\right]\\ \; & \leq Ce^{-cn}. \end{array}$$

By Theorem 1, it follows that

(84) $$\left|\log\frac{P(T_{n}^{H}>x)}{1-\Phi (x)}\right|\leq Ce^{-cn},\;0\leq x<h_{n}.$$

Using $|e^{u}-1|\leq |u|e^{\left|u\right|}$ in (84), we obtain

(85) $$\begin{array}{cc} H_{2} & =\underset{0\leq x<h_{n}}{\sup}\left|P\left(T_{n}^{H}>x\right)-\left\{1-\Phi \left(x\right)\right\}\right|\\ \; & \leq Ce^{-cn}\underset{x\geq 0}{\sup}\left\{1-\Phi \left(x\right)\right\}\leq Ce^{-cn}. \end{array}$$

Similarly, the left-tail estimate in Theorem 1 together with $|e^{u}-1|\leq |u|e^{\left|u\right|}$ gives

(86) $$\begin{array}{cc} H_{3} & \leq Ce^{-cn}\underset{x\leq 0}{\sup}\Phi \left(x\right)\leq Ce^{-cn}. \end{array}$$

Combining (82), (85), (86), and (83), we obtain (10). □

### 5.8. Proofs of Corollaries 4 and 5

For each fixed y>0, Theorem 3 and the normal tail asymptotics give

$$\lim_{n\to \infty }\frac{1}{a_{n}^{2}}\log P\left(\pm T_{n}^{H}\geq a_{n}y\right)=-\frac{y^{2}}{2}.$$

The argument for Borel sets in the proof of Corollary 1 proves (18). For (19), let $c_{0}$ be the constant in Theorem 3 and put $h_{n}=c_{0}\sqrt{n}/2$. Repeating the four estimates in the proof of Corollary 2 with this theorem gives the result for any $0<c<\min\{c_{0},c_{0}^{2}/8\}$.

## 6. Discussion

We have established relative error bounds for the normal approximation to the Studentized Harris estimator under a finite (2+ρ)th moment assumption. Theorems 1 and 3 treat $p_{0}=0$ with $p_{1}>0$ and $p_{1}=0$, respectively. Theorem 2 allows $p_{0}>0$ and gives the corresponding estimates conditional on non-extinction and on survival up to the observation generation, without requiring $p_{1}>0$. The moderate deviation principles, Berry–Esseen bounds, and confidence intervals follow under the respective probability measures.

For the cases without extinction, the proof applies an independent self-normalized moderate deviation theorem and compares the resulting quadratic sum with $V_{n}^{2}$. The population lower bound is deterministic in the Böttcher case. For $p_{0}>0$, the proof uses a Berry–Esseen bound for the descendant totals under P. A population lower-tail bound and a conditional mixture identity control survival conditioning; no independence of the original descendant trees is asserted under $P_{S}$. The same Studentization identity applies in all three cases.

The confidence interval requires the sum of squared offspring numbers in addition to generation sizes. Its coverage is justified asymptotically; the constants do not yield a computable finite-sample coverage bound. The numerical experiments concern $p_{0}=0$, $p_{1}\in \left(0,1\right)$; finite-generation performance under survival conditioning and in the Böttcher case is not examined here. A corresponding result based only on generation sizes would also require further study.

## Figures and Tables

**Figure 1:**
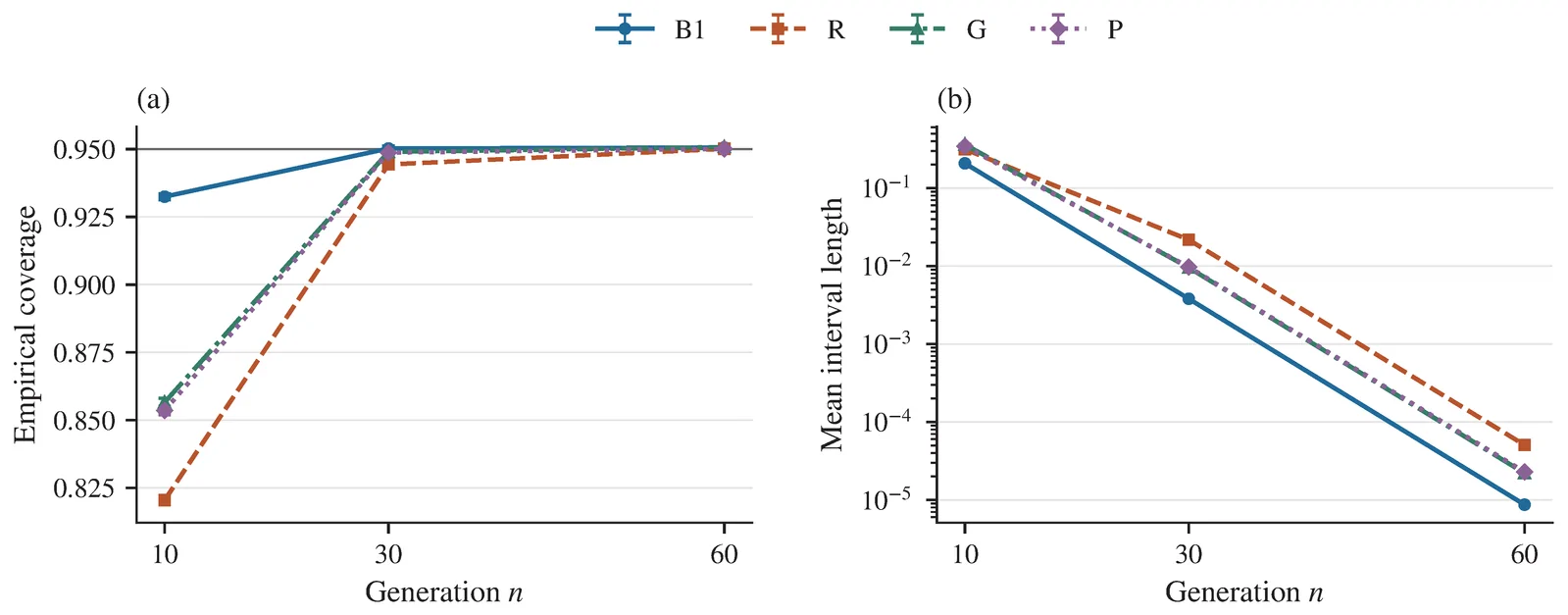
Empirical coverage (**a**) and mean length (**b**) of the nominal 95% interval for B1, R, G, and P at n=10,30,60. Each model uses 200,000 independent trees. Coverage bars are pointwise 95% Wilson intervals; length bars extend 1.96 Monte Carlo standard errors from the mean. The horizontal line in (**a**) marks 0.95. Panel (**b**) uses a logarithmic vertical axis. Lines connect the three recorded estimates.

**Figure 2:**
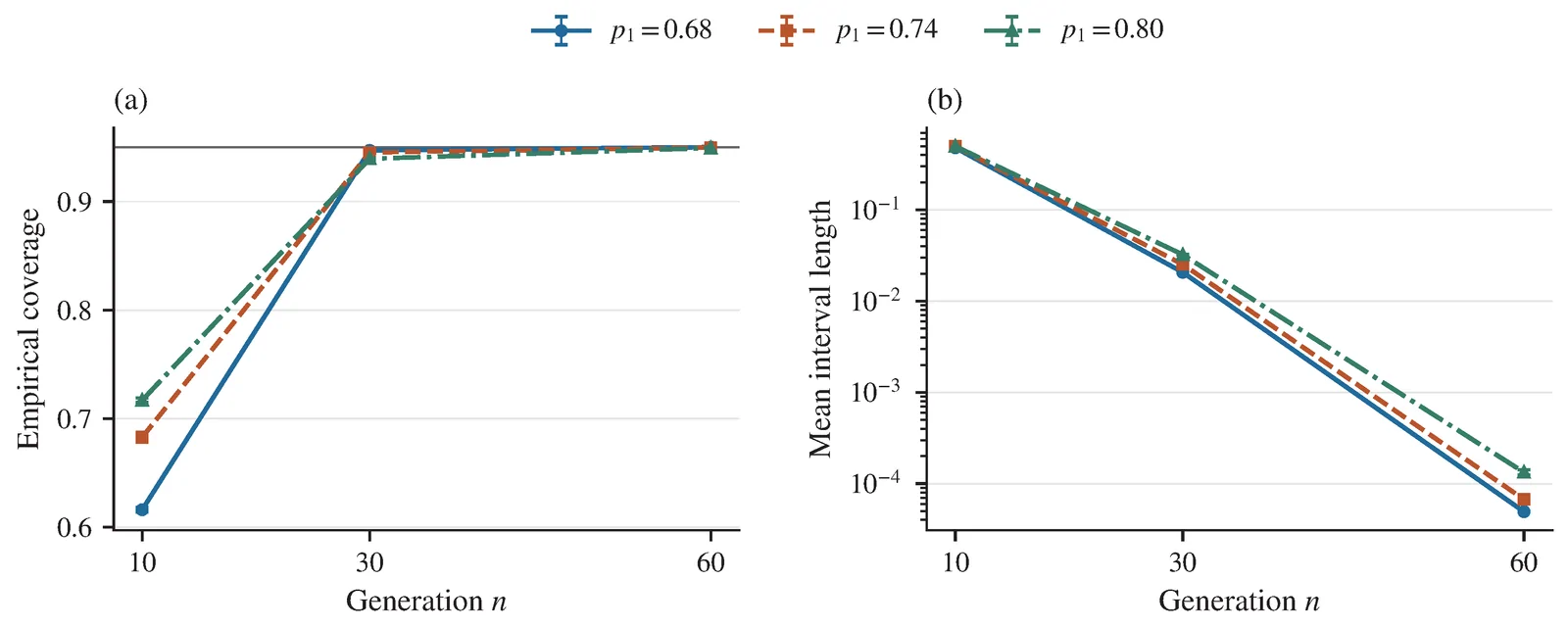
Empirical coverage (**a**) and mean interval length (**b**) for S68, S74, and S80 at n=10,30,60. The laws have common mean m=1.5 and variance $\sigma^{2}=2$, with $p_{1}=0.68,0.74,0.80$, respectively. Each law uses 200,000 independent trees. Error bars are defined as in Figure 1; the length axis is logarithmic.

**Figure 3:**
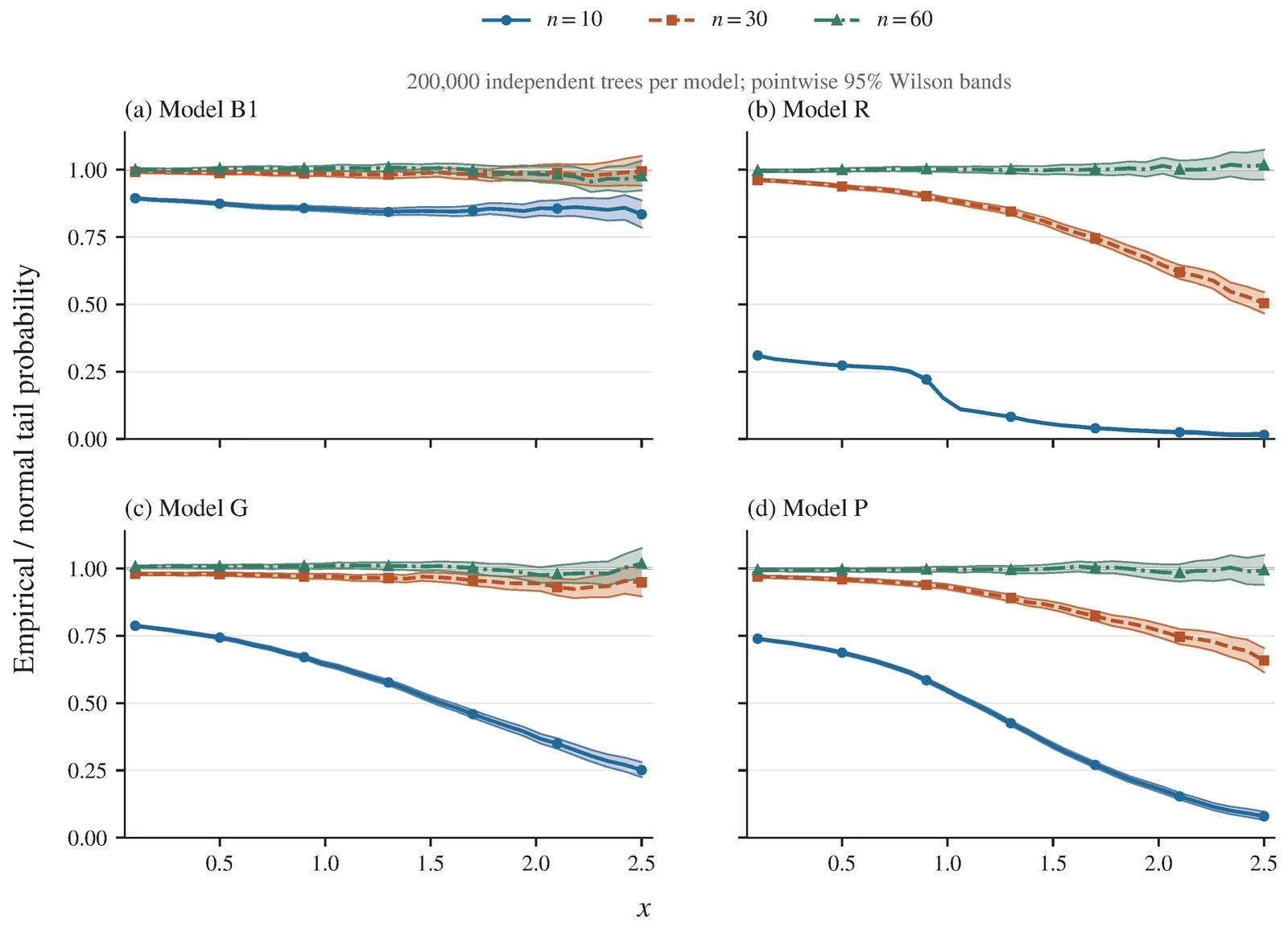
Right-tail probability ratios for B1, R, G, and P at n=10,30,60, based on 200,000 independent trees per model. Shaded bands, bounded by thin lines, are pointwise 95% Wilson intervals divided by 1−Φ(x); the horizontal line marks 1. Colors, line styles, and markers identify the same generations in every panel.

**Figure 4:**
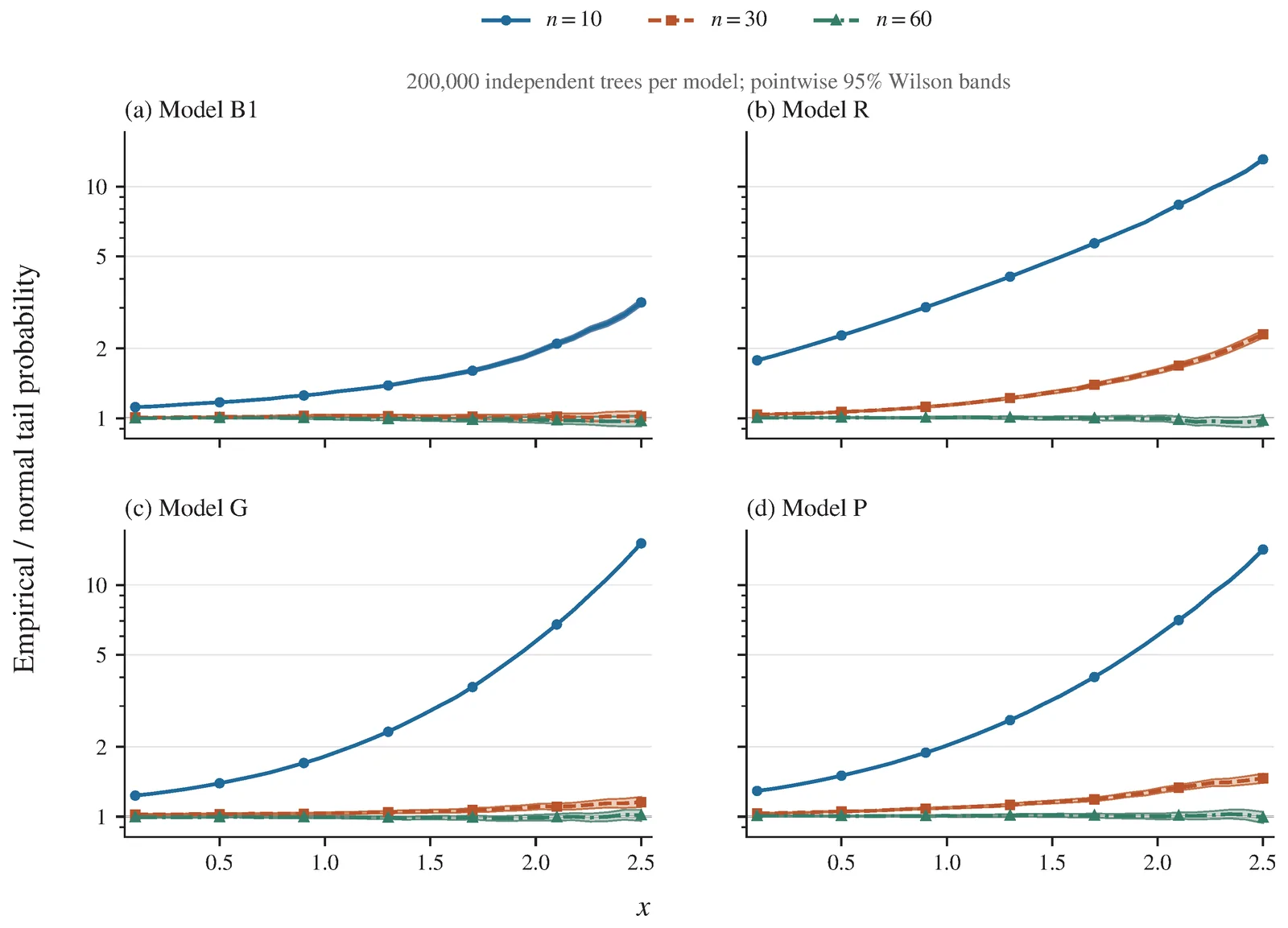
Left-tail probability ratios for the models and generations in Figure 3. Shaded bands, bounded by thin lines, are pointwise 95% Wilson intervals divided by Φ(−x). The vertical axis is logarithmic, and the horizontal line marks 1.

**Table 1:** Offspring laws; for P, the third moment is infinite.

Model	$p_{1}$	*m*	$\sigma^{2}$	Skewness
B1	0.5000	1.50	0.2500	0.000
B2	0.4000	1.60	0.2400	−0.408
B3	0.3000	2.10	0.6900	−0.188
B4	0.2000	2.10	0.4900	−0.140
H	0.9500	1.50	4.7500	4.129
R	0.5500	1.50	5.2500	41.357
G	0.6667	1.50	0.7500	2.309
P	0.6170	1.50	1.0009	+∞
S68	0.6800	1.50	2.0000	5.842
S74	0.7400	1.50	2.0000	5.418
S80	0.8000	1.50	2.0000	4.994

**Table 2:** Performance of the nominal 95% interval. Brackets are pointwise 95% Wilson intervals; the last column is the Monte Carlo standard error of mean length.

Model	*n*	Coverage	Wilson Interval	Mean Length	MC s.e.
B1	10	0.9324	[0.9313, 0.9335]	0.20787	$1.56\times 10^{-4}$
B1	30	0.9502	[0.9493, 0.9512]	0.00380	$4.41\times 10^{-6}$
B1	60	0.9506	[0.9496, 0.9515]	$8.69\times 10^{-6}$	$1.01\times 10^{-8}$
R	10	0.8205	[0.8188, 0.8221]	0.31529	0.00141
R	30	0.9443	[0.9433, 0.9453]	0.02172	$3.16\times 10^{-5}$
R	60	0.9501	[0.9491, 0.9510]	$5.06\times 10^{-5}$	$9.11\times 10^{-8}$
G	10	0.8566	[0.8550, 0.8581]	0.36195	$3.68\times 10^{-4}$
G	30	0.9490	[0.9480, 0.9499]	0.00955	$2.68\times 10^{-5}$
G	60	0.9508	[0.9498, 0.9517]	$2.21\times 10^{-5}$	$8.22\times 10^{-8}$
P	10	0.8536	[0.8520, 0.8551]	0.34505	$4.46\times 10^{-4}$
P	30	0.9487	[0.9477, 0.9497]	0.00973	$2.14\times 10^{-5}$
P	60	0.9500	[0.9491, 0.9510]	$2.28\times 10^{-5}$	$5.71\times 10^{-8}$

**Table 3:** Comparison at n=60 on identical paths. The difference is estimated-variance coverage minus known-variance coverage, with its paired Monte Carlo standard error in parentheses.

Model	Coverage	Oracle	Difference (s.e.)	Mean Length
B1	0.9506	0.9506	0.0000 (0.0000)	$8.69\times 10^{-6}$
H	0.8963	0.9520	−0.0558 (0.0005)	0.02433
R	0.9501	0.9501	0.0000 (0.0000)	$5.06\times 10^{-5}$
G	0.9508	0.9508	0.0000 (0.0000)	$2.21\times 10^{-5}$
P	0.9500	0.9502	−0.0001 (0.0001)	$2.28\times 10^{-5}$
S68	0.9501	0.9501	0.0000 (0.0000)	$4.93\times 10^{-5}$
S74	0.9497	0.9497	0.0000 (0.0000)	$6.71\times 10^{-5}$
S80	0.9493	0.9493	0.0000 (0.0000)	$1.33\times 10^{-4}$

**Table 4:** Tail ratios at x=2.5 and pointwise 95% Wilson intervals.

Model	*n*	Right Ratio	Wilson Interval	Left Ratio	Wilson Interval
B1	10	0.834	[0.785, 0.886]	3.162	[3.066, 3.261]
B1	30	0.994	[0.941, 1.051]	1.018	[0.963, 1.075]
B1	60	0.977	[0.923, 1.033]	0.974	[0.921, 1.031]
R	10	0.015	[0.010, 0.024]	13.109	[12.917, 13.303]
R	30	0.504	[0.466, 0.545]	2.306	[2.224, 2.391]
R	60	1.017	[0.963, 1.074]	0.978	[0.924, 1.034]
G	10	0.251	[0.225, 0.281]	15.115	[14.911, 15.322]
G	30	0.949	[0.896, 1.004]	1.155	[1.097, 1.217]
G	60	1.018	[0.963, 1.075]	1.013	[0.959, 1.070]
P	10	0.080	[0.065, 0.097]	14.223	[14.024, 14.425]
P	30	0.658	[0.614, 0.704]	1.464	[1.398, 1.532]
P	60	0.993	[0.939, 1.050]	0.992	[0.938, 1.049]

## Data Availability

No external datasets were analyzed. The accompanying Python (Python 3.14.8) code reproduces the numerical study. The supplementary output includes model parameters, tail probabilities, coverage and length summaries, and run metadata.

## Supplementary Materials

The following supporting information can be downloaded at: https://www.mdpi.com/article/10.3390/e28101145/s1.
