# Supplementary material for: Self-Normalized Cramér-Type Moderate Deviations for the Harris Estimator of a Supercritical Branching Process
Source: Entropy (Basel). 2026 Oct 18;28(10):1145. doi: 10.3390/e28101145 (PMC13703742; doi:10.3390/e28101145)
Supplement: Supplementary file 1 [file entropy-28-01145-s001.zip › simulation_results/coverage_and_length.pdf]

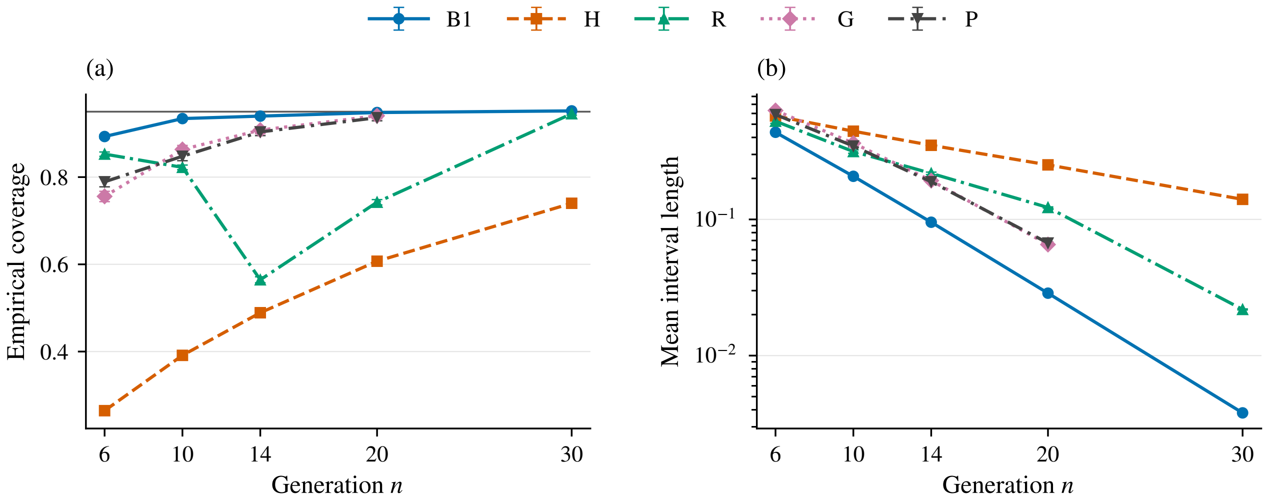

B1
H
R
G
P
(a)
(b)
Empirical coverage
0.8
0.6
0.4
6
10
14
20
30
Generation n
Mean interval length
10^{-1}
10^{-2}
6
10
14
20
30
Generation n
