# Supplementary material for: Self-Normalized Cramér-Type Moderate Deviations for the Harris Estimator of a Supercritical Branching Process
Source: Entropy (Basel). 2026 Oct 18;28(10):1145. doi: 10.3390/e28101145 (PMC13703742; doi:10.3390/e28101145)
Supplement: Supplementary file 1 [file entropy-28-01145-s001.zip › simulation_results/expanded_left_tails.pdf]

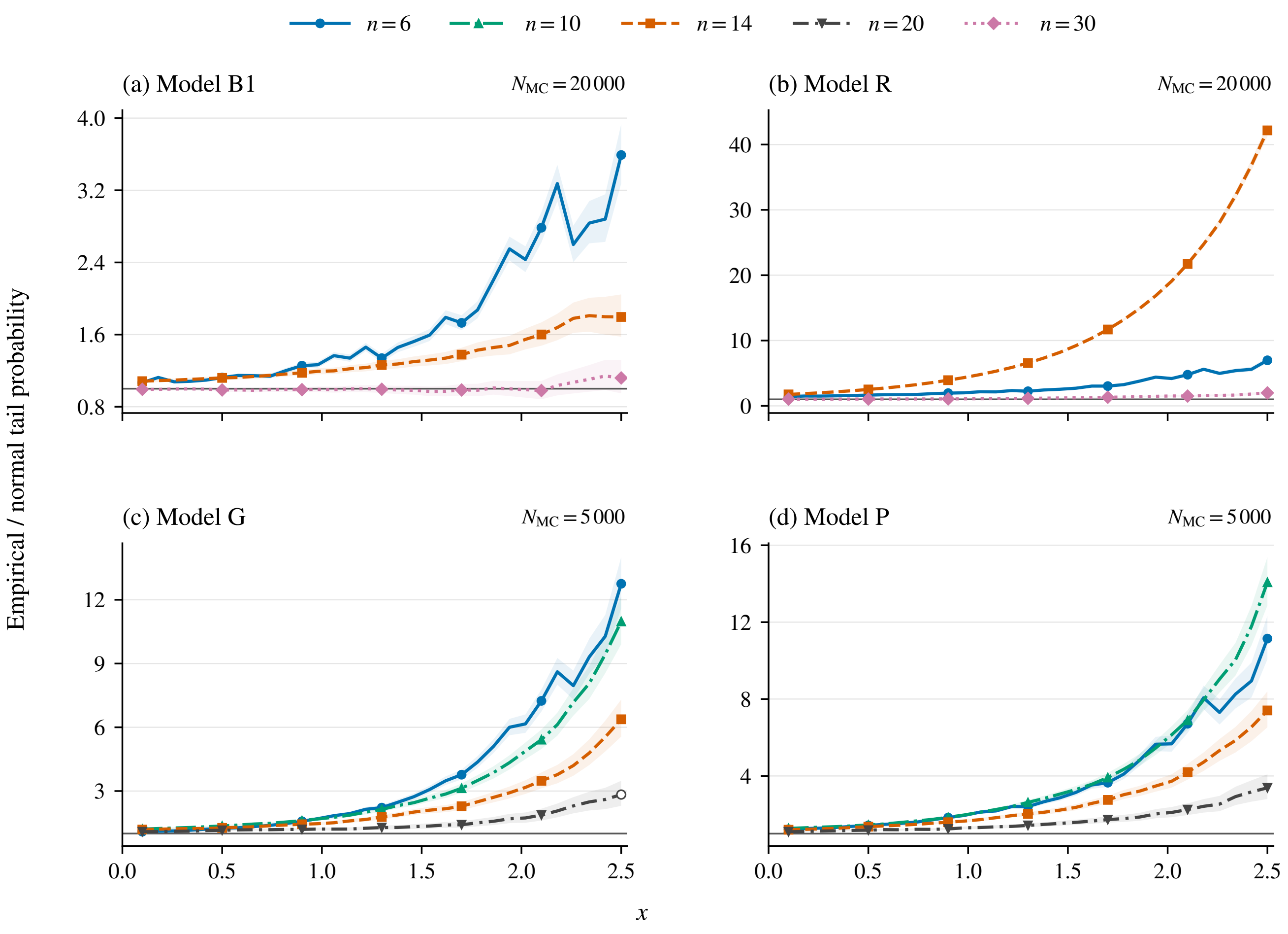

$n = 6$
$n = 10$
$n = 14$
$n = 20$
$n = 30$
(a) Model B1
$N_{MC} = 20\,000$
(b) Model R
$N_{MC} = 20\,000$
(c) Model G
$N_{MC} = 5\,000$
(d) Model P
$N_{MC} = 5\,000$
Empirical / normal tail probability
x
