# Supplementary material for: Self-Normalized Cramér-Type Moderate Deviations for the Harris Estimator of a Supercritical Branching Process
Source: Entropy (Basel). 2026 Oct 18;28(10):1145. doi: 10.3390/e28101145 (PMC13703742; doi:10.3390/e28101145)
Supplement: Supplementary file 1 [file entropy-28-01145-s001.zip › simulation_results/expanded_right_tails.pdf]

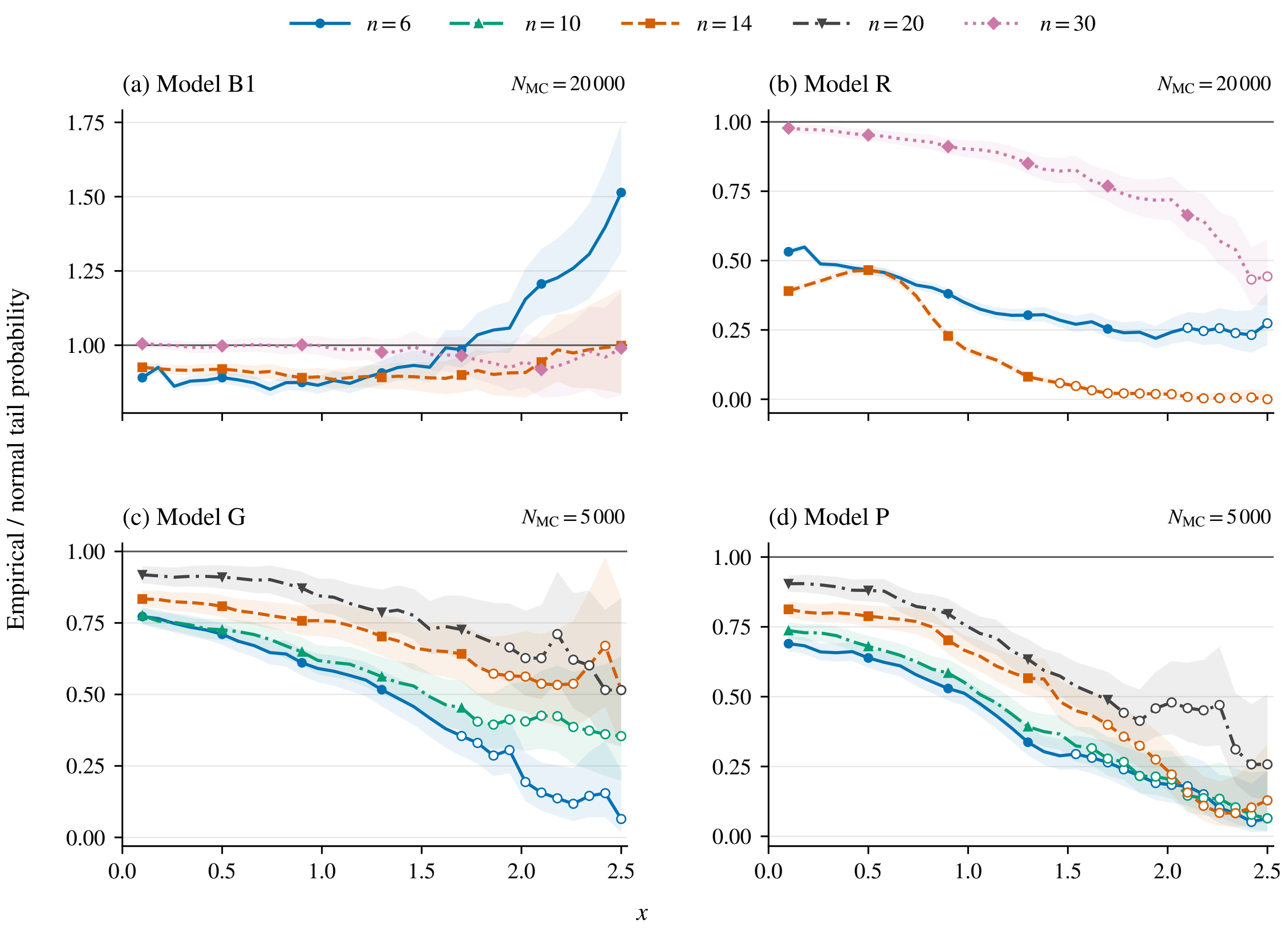

n = 6
n = 10
n = 14
n = 20
n = 30
(a) Model B1
N_MC = 20 000
(b) Model R
N_MC = 20 000
(c) Model G
N_MC = 5 000
(d) Model P
N_MC = 5 000
Empirical / normal tail probability
x
