# Supplementary material for: Self-Normalized Cramér-Type Moderate Deviations for the Harris Estimator of a Supercritical Branching Process
Source: Entropy (Basel). 2026 Oct 18;28(10):1145. doi: 10.3390/e28101145 (PMC13703742; doi:10.3390/e28101145)
Supplement: Supplementary file 1 [file entropy-28-01145-s001.zip › simulation_results/original_models_right_tails.pdf]

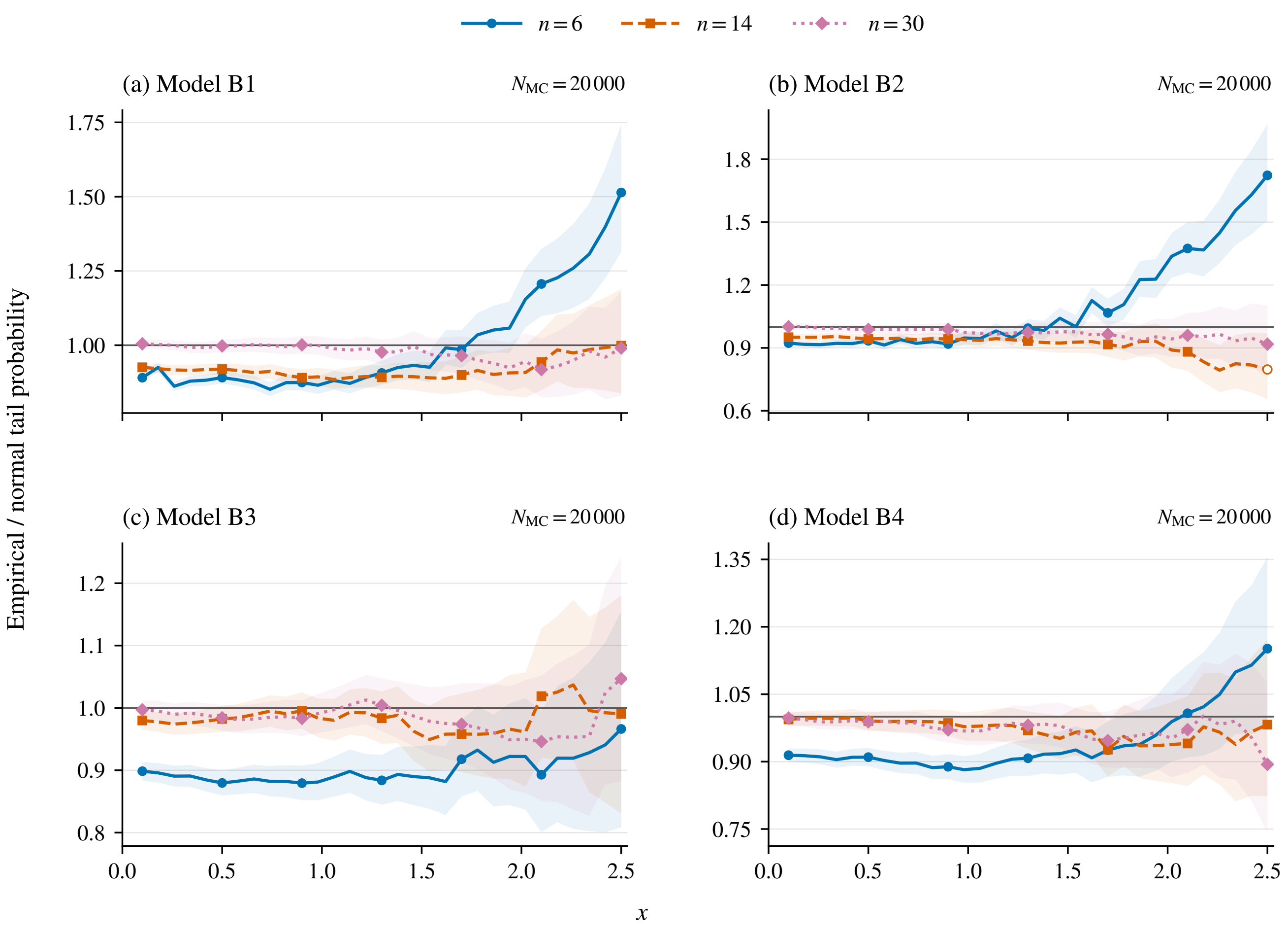

$n = 6$
$n = 14$
$n = 30$
(a) Model B1
$N_{MC} = 20\,000$
1.75
1.50
1.25
1.00
(b) Model B2
$N_{MC} = 20\,000$
1.8
1.5
1.2
0.9
0.6
(c) Model B3
$N_{MC} = 20\,000$
1.2
1.1
1.0
0.9
0.8
(d) Model B4
$N_{MC} = 20\,000$
1.35
1.20
1.05
0.90
0.75
0.0
0.5
1.0
1.5
2.0
2.5
Empirical / normal tail probability
x
