# Supplementary material for: Self-Normalized Cramér-Type Moderate Deviations for the Harris Estimator of a Supercritical Branching Process
Source: Entropy (Basel). 2026 Oct 18;28(10):1145. doi: 10.3390/e28101145 (PMC13703742; doi:10.3390/e28101145)
Supplement: Supplementary file 1 [file entropy-28-01145-s001.zip › simulation_results/p1_sensitivity.pdf]

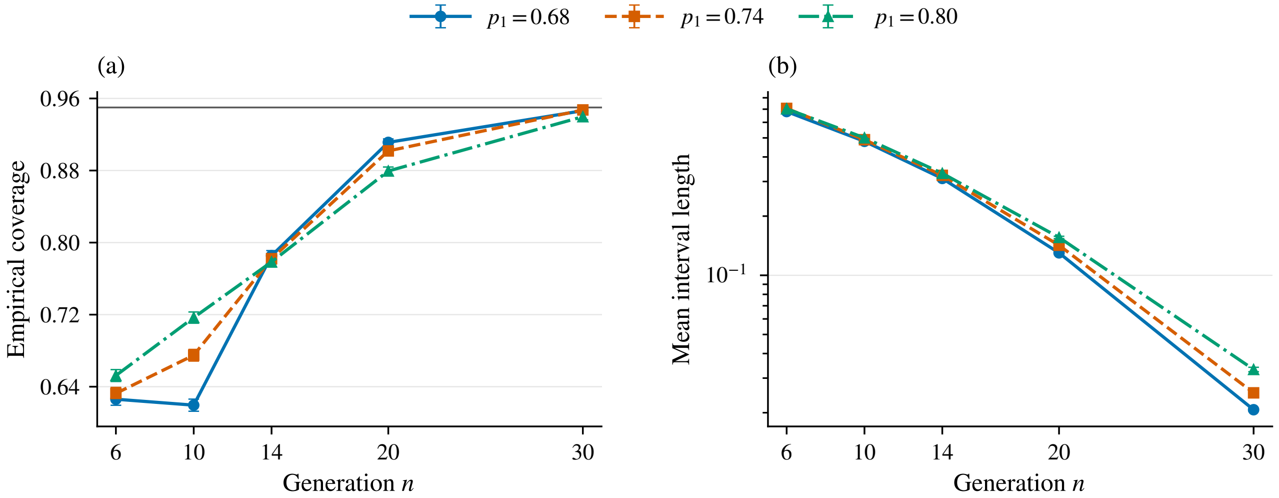

$p_1 = 0.68$
$p_1 = 0.74$
$p_1 = 0.80$
(a)
0.96
0.88
0.80
0.72
0.64
Empirical coverage
6
10
14
20
30
Generation n
(b)
10^{-1}
Mean interval length
6
10
14
20
30
Generation n
