# Supplementary material for: Self-Normalized Cramér-Type Moderate Deviations for the Harris Estimator of a Supercritical Branching Process
Source: Entropy (Basel). 2026 Oct 18;28(10):1145. doi: 10.3390/e28101145 (PMC13703742; doi:10.3390/e28101145)
Supplement: Supplementary file 1 [file entropy-28-01145-s001.zip › simulation_results/simulation_comparison_table.pdf]

Table caption. Comparison on the same simulated paths. The known-variance interval is an oracle benchmark. The difference equals estimated-variance coverage minus oracle coverage.

Model	n	Coverage	Oracle	Difference (s.e.)	Length	Oracle length
B1	30	0.9518	0.9518	0.0000 (0.0000)	0.0038	0.0038
H	30	0.7400	0.9611	-0.2212 (0.0030)	0.1404	0.4814
R	30	0.9453	0.9531	-0.0078 (0.0011)	0.0218	0.0221
G	20	0.9406	0.9496	-0.0090 (0.0017)	0.0654	0.0679
P	20	0.9362	0.9614	-0.0252 (0.0030)	0.0671	0.0739
