# Supplementary material for: Self-Normalized Cramér-Type Moderate Deviations for the Harris Estimator of a Supercritical Branching Process
Source: Entropy (Basel). 2026 Oct 18;28(10):1145. doi: 10.3390/e28101145 (PMC13703742; doi:10.3390/e28101145)
Supplement: Supplementary file 1 [file entropy-28-01145-s001.zip › simulation_results/simulation_coverage_table.pdf]

Table caption. Empirical performance of the nominal 95% interval. Coverage brackets are pointwise 95%Wilson intervals. The last column gives the Monte Carlo standard error of mean length.

Model	n	N_{MC}	Coverage	Wilson interval	Mean length	MC s.e.
B1	6	20000	0.8931	[0.8887, 0.8973]	0.4363	0.000775
B1	14	20000	0.9398	[0.9365, 0.9431]	0.0954	0.000287
B1	30	20000	0.9518	[0.9488, 0.9547]	0.0038	0.000014
H	6	20000	0.2645	[0.2584, 0.2706]	0.5749	0.009318
H	14	20000	0.4890	[0.4821, 0.4959]	0.3502	0.004131
H	30	20000	0.7400	[0.7339, 0.7460]	0.1404	0.001916
R	6	20000	0.8528	[0.8478, 0.8576]	0.5260	0.009287
R	14	20000	0.5645	[0.5577, 0.5714]	0.2179	0.002334
R	30	20000	0.9453	[0.9421, 0.9484]	0.0218	0.000101
G	6	5000	0.7558	[0.7437, 0.7675]	0.6306	0.004813
G	14	5000	0.9078	[0.8995, 0.9155]	0.1927	0.001516
G	20	5000	0.9406	[0.9337, 0.9468]	0.0654	0.000715
P	6	5000	0.7892	[0.7777, 0.8003]	0.5885	0.005157
P	14	5000	0.9036	[0.8951, 0.9115]	0.1891	0.001569
P	20	5000	0.9362	[0.9291, 0.9426]	0.0671	0.000701
