# Supplementary material for: Self-Normalized Cramér-Type Moderate Deviations for the Harris Estimator of a Supercritical Branching Process
Source: Entropy (Basel). 2026 Oct 18;28(10):1145. doi: 10.3390/e28101145 (PMC13703742; doi:10.3390/e28101145)
Supplement: Supplementary file 1 [file entropy-28-01145-s001.zip › simulation_results/simulation_model_table.pdf]

Table caption. Offspring laws in the simulation study. The three sensitivity laws share $m = 1.5$ and $\sigma^2 = 2$. For P, $+\infty$ indicates an infinite third moment.

Model	p_1	m	σ^2	Skewness
B1	0.5000	1.50	0.2500	0.000
B2	0.4000	1.60	0.2400	-0.408
B3	0.3000	2.10	0.6900	-0.188
B4	0.2000	2.10	0.4900	-0.140
H	0.9500	1.50	4.7500	4.129
R	0.5500	1.50	5.2500	41.357
G	0.6667	1.50	0.7500	2.309
P	0.6170	1.50	1.0009	$+\infty$
S68	0.6800	1.50	2.0000	5.842
S74	0.7400	1.50	2.0000	5.418
S80	0.8000	1.50	2.0000	4.994
