# Supplementary material for: Self-Normalized Cramér-Type Moderate Deviations for the Harris Estimator of a Supercritical Branching Process
Source: Entropy (Basel). 2026 Oct 18;28(10):1145. doi: 10.3390/e28101145 (PMC13703742; doi:10.3390/e28101145)
Supplement: Supplementary file 1 [file entropy-28-01145-s001.zip › simulation_results/simulation_sensitivity_table.pdf]

Table caption. Sensitivity to p_1 at nominal coverage 95%, with $m = 1.5$ and $\sigma^2 = 2$. Coverage brackets are pointwise 95% Wilson intervals. The last column gives the Monte Carlo standard error of mean length.

Model	n	N_{MC}	Coverage	Wilson interval	Mean length	MC s.e.
S68	6	20000	0.6261	[0.6194, 0.6328]	0.6807	0.004943
S68	14	20000	0.7854	[0.7797, 0.7911]	0.3109	0.001403
S68	30	20000	0.9464	[0.9432, 0.9494]	0.0207	0.000185
S74	6	20000	0.6325	[0.6258, 0.6392]	0.7064	0.004983
S74	14	20000	0.7821	[0.7763, 0.7878]	0.3225	0.001533
S74	30	20000	0.9473	[0.9441, 0.9503]	0.0252	0.000290
S80	6	20000	0.6524	[0.6458, 0.6590]	0.7029	0.005108
S80	14	20000	0.7785	[0.7727, 0.7843]	0.3302	0.001745
S80	30	20000	0.9397	[0.9364, 0.9430]	0.0332	0.000438
